# Dual-metallic porphyrinic MOFs with pH/ROS-responsive release for antibacterial and antioxidant therapy of diabetic infected wounds

**DOI:** 10.7150/thno.135365

**Published:** 2026-07-29

**Authors:** Yipeng Pang, Fructueux Modeste Amona, Hanyuan Liu, Shuming Tang, Jianlong Lao, Xi Chen, Xiaoye Wang

**Affiliations:** 1 College of Animal Science and Technology, Guangxi University; Guangxi Key Laboratory of Animal Reproduction, Breeding and Disease Control; Guangxi Zhuang Autonomous Region Engineering Research Center of Veterinary Biologics, Nanning, 530004, Guangxi, China.; 2 Institute of Cellular and Molecular Biology, School of Life Science, Jiangsu Normal University, Xuzhou, 221116, Jiangsu, China.

**Keywords:** dual-metallic porphyrinic MOFs, diabetic wound, multidrug-resistant infections, antioxidant, stimuli-responsive, redox homeostasis

## Abstract

**Rationale:**

Chronic diabetic wounds are complicated by multidrug-resistant (MDR) infections and a deleterious oxidative microenvironment, rendering single-target therapies ineffective. This study aimed to develop a smart, dual-metal-organic framework (MOF) nanoplatform (PCNZnCy) capable of microenvironment-responsive, coordinated antibacterial, antioxidant, and pro-regenerative therapy for infected diabetic wounds.

**Methods:**

The nanoplatform PCNZnCy was constructed by integrating the natural flavonoid chrysin into a zinc-doped porphyrinic MOF (PCN-224), enabling pH- and reactive oxygen species (ROS)-triggered co-release of Zn²⁺ and chrysin. Antibacterial and anti-biofilm activity was assessed against biofilm-embedded MDR pathogens, including methicillin-resistant *Staphylococcus aureus* (MRSA) and *Pseudomonas aeruginosa* (PA). Antioxidant and signaling effects were evaluated by measuring ROS scavenging, mitochondrial function, endothelial cell proliferation, and activation of Nrf2/HO-1, STAT3, and Akt pathways, with confirmation using the Nrf2 inhibitor ML385. To assess efficacy and safety in a clinically meaningful setting, we used a diabetic murine wound model infected with *P. aeruginosa* and a systems-level transcriptomic analysis to capture the broader tissue response.

**Results:**

We found that PCNZnCy showed strong synergistic antibacterial activity against MRSA and PA. Beyond that, it scavenged excess ROS, triggered Nrf2/HO-1 signaling, modulated STAT3 and Akt signaling, preserved mitochondrial health, and spurred endothelial cell growth. In diabetic mice, wounds healed faster, with improved collagen deposition and increased formation of new blood vessels, while the compound remained safe. RNA-seq data underscored a decisive move from pro-inflammatory to pro-regenerative gene expression.

**Conclusions:**

PCNZnCy adapts to the wound's specific microenvironment, enabling it to kill bacteria, reduce oxidative stress, and promote tissue regeneration. In the infected diabetic models, it worked consistently well. This makes it a strong candidate for multifactorial, complex wound pathologies, in which multiple complications overlap.

## Introduction

The worldwide clinical and economic burden of chronic diabetic wounds is steep, with patients enduring a profoundly diminished quality of life 1. Rather than progressing through the ordered repair process, these lesions stall in a hostile microenvironment where persistent bacteria, unrelenting oxidative stress, and dysregulated signaling arrest endogenous healing mechanisms 2. Moreover, multidrug-resistant strains such as methicillin-resistant *Staphylococcus aureus* (MRSA) and *Pseudomonas aeruginosa* (PA), identified by the WHO 3,4, often form persistent biofilms which are resistant to conventional drugs and escape detection by the immune system 5. In addition, a large number of reactive oxygen species (ROS) not only damage the adjacent tissues but also inhibit the growth of new blood vessels and promote continued inflammation, thereby hindering effective regeneration. Therefore, single treatments are not sufficient; medical personnel should adopt coordinated and multi-faceted approaches to restore the local equilibrium.

Effective diabetic wound treatment demands more than accelerated closure; it must also fend off infection, activate the host's intrinsic healing cascade, recapitulate key extracellular matrix (ECM) cues, retain moisture, and limit eventual scarring 6,7. Furthermore, studies on antibacterial materials have advanced from conventional antibiotic therapies to surface modifications that prevent the formation of biofilms without causing resistance 8,9. Nanozyme-integrated scaffolds have also been found advantageous for the poor oxidative state of chronic wounds and combat infections by biofilms 10,11. Consequently, metal-organic frameworks (MOFs) have received considerable attention owing to their large surface areas, variable pore sizes and chemically adjustable properties, which make them suitable carriers for drug delivery and protection 12,13. Some MOFs behave like natural oxidoreductases or release antibacterial metal ions, acting as built-in nanozymes or reserve sources 14. However, it is still challenging to establish a uniform MOF system to handle the severe oxidative stress and delayed tissue repair in diabetic wounds. The MOF systems currently reported, such as ZIF-8 15,16, UiO-66 17 and composites like CD@H–MnO₂ and CD/Zr-MOF/MnO₂-x 18, are usually activated in the acidic environment of infected wounds. Nevertheless, their activities and efficiencies decline after several catalytic cycles due to the destruction of their structures. Therefore, bimetallic MOF networks are regarded as a potential method to solve these problems 14.

Porphyrinic MOFs, an essential subclass of MOFs, are dual-metal MOF networks from porphyrin linkers and metal ions/clusters. Recently, PCN-224 has been explored as a photosensitizer for photodynamic antibacterial therapy (PDT) and as a nanomaterial for antibacterial treatment 19-21, demonstrating excellent biocompatibility and cellular uptake due to its size and spherical shape 22. Unlike other dual-metal MOFs such as multifunctional genipin (GP)-crosslinked chitosan (CS) -based hydrogels decorated with the biomimetic MOF-nanozymes and the natural antibacterial agent chlorogenic acid (CGA), which is named MCGC 14, the zinc (Zn)-doped porphyrinic metal-organic framework (PCN-224 MOF), with zirconium (Zr) clusters linked by carboxylated porphyrin, is beneficial for wound management. Incorporation of Zn, vital for immune function, cell proliferation, wound healing, and DNA synthesis, enhances the framework's therapeutic potential, making PCN-224 MOFs a promising nanoplatform for multi-drug delivery against complex diseases, such as diabetic-infected wounds. Diabetic wounds, characterized by immune dysfunction, high glucose, and moisture, are prone to severe bacterial infections, acidosis, and elevated ROS levels that perpetuate inflammation 23. Relying on antibiotics is concerning due to their overuse and the emergence of MDR pathogens 24, underscoring the urgent need for safe, natural compound-based alternatives. While Zn-doped PCN-224 shows some antibacterial activity 25, it lacks dual-metal synergy and triggered release of metal ions and flavonoids. Earlier MOF-flavonoid composites rely on monometallic frameworks and simple adsorption, with limited responsiveness to infected wounds. To address these gaps, a designed bimetallic (Zr/Zn) porphyrinic MOF co-loaded with chrysin could advance conventional Zn-doped PCN-224 or flavonoid-MOF hybrids, providing combined antibacterial, antioxidant, and wound-healing functions in a single, stimuli-responsive platform for the management of complex MDR-infected diabetic wounds. Unlike other natural flavonoids, Chrysin (5,7-dihydroxyflavone), found in plants and honey, has robust antimicrobial activity against both Gram-positive and Gram-negative pathogens, as well as anti-inflammatory and antioxidant properties, promotes keratinocyte differentiation, and accelerates healing 26,27. It has recently been investigated for treating wounds infected by PA and *S. aureus* 26 or for wound-healing applications 28. Additionally, Chrysin-functionalized PCN-224 MOFs offer a highly versatile nanoplatform to address complex diabetic wound pathology.

**Scheme 1:**
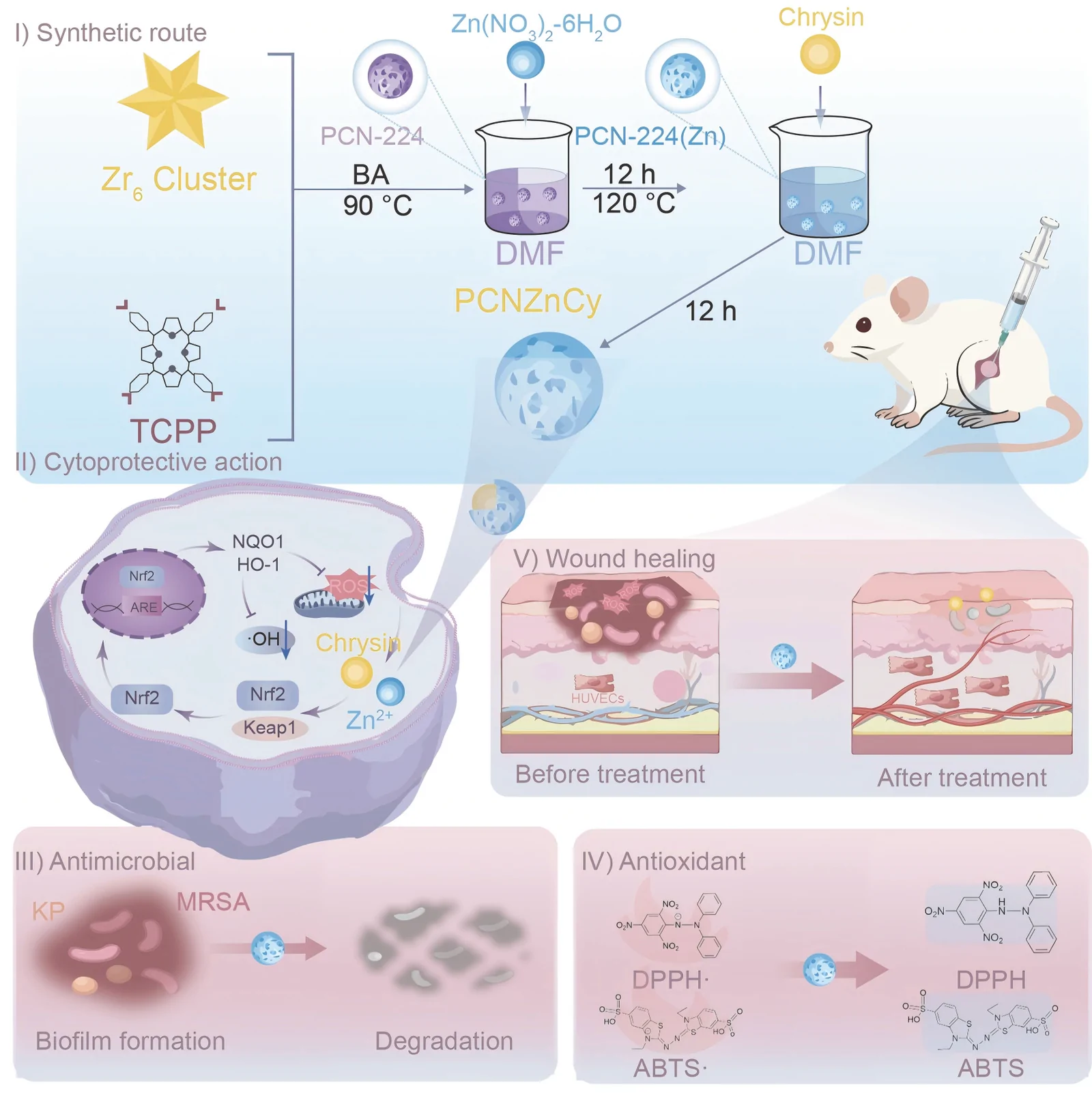
** Schematic representation of the synthesis and mechanism of PCNZnCy for diabetic wound treatment. I)** Synthetic route, self-assembly of PCN-224 using a tetrakis(4-carboxyphenyl)porphyrin (TCPP) and Zr_6_ clusters to produce PCN-224(Zn), then loading chrysin to form dual-metallic porphyrinic MOF loaded with Chrysin (PCNZnCy). BA denotes benzoic acid. **II)** Mechanism of antioxidant and cytoprotective action: PCN-ZnCy enters cells, scavenges ROS, disrupts the Keap1–Nrf2 complex to activate Nrf2/HO-1, upregulates ARE genes, reduces oxidative stress in human umbilical vein endothelial cells (HUVECs), and promotes proliferation and angiogenesis. III) Antimicrobial, IV) antioxidant, and V) therapeutic effects showing wound healing by improving angiogenesis, collagen deposition, and re-epithelialization in oxidative environments.

Herein, this work developed PCNZnCy, a smart nanoplatform constructed by loading chrysin into a zinc-doped porphyrinic MOF (PCN-224) to respond to the wound microenvironmental pH and ROS, thereby enabling controlled delivery of chrysin and Zn²⁺ ions (Scheme 1). PCNZnCy effectively disrupts the diabetic wound cycle by eradicating MDR pathogens and biofilms, scavenging ROS to reduce oxidative damage via the Nrf2/HO-1 pathway, and promoting endothelial proliferation and angiogenesis. In a clinically relevant murine PA-infected diabetic wound model, PCNZnCy accelerated wound closure, enhanced mature collagen deposition and neovascularization, and demonstrated high biosafety. This study offers a nanotherapeutic strategy for diabetic-infected wounds, as demonstrated in a PA-infected diabetic murine model, though broader applications to other disease types or polymicrobial infections may require further investigation.

## Results and Discussion

### Fabrication and characterization of PCNZnCy

**Figure 1:**
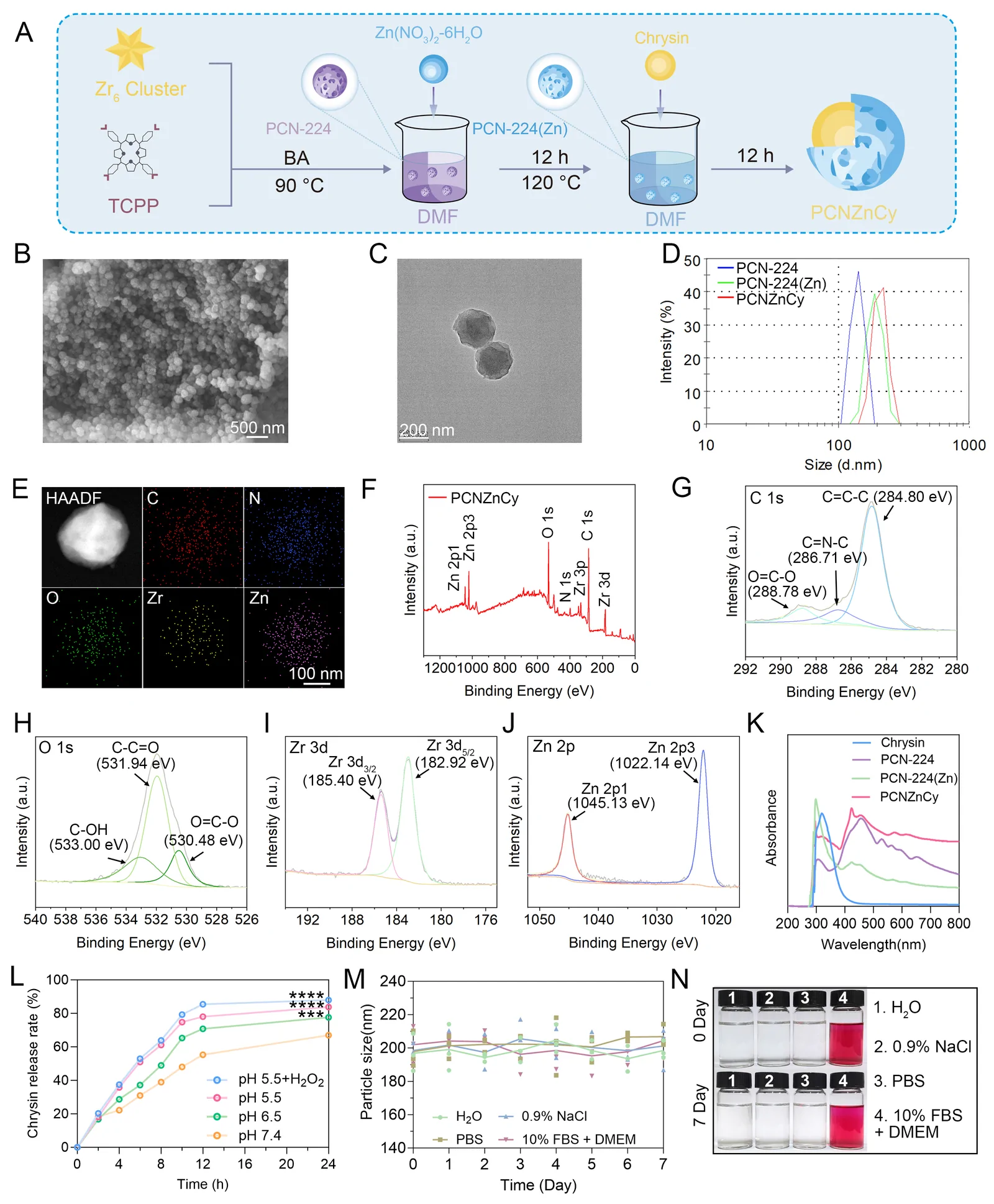
** Fabrication and characterization of PCNZnCy. (A)** Schematic illustration of the stepwise synthesis of PCNZnCy. **(B, C)** SEM and TEM images of PCNZnCy. Scale bars: 500 nm and 200 nm. **(D)** DLS analysis showing the hydrodynamic diameter and size distribution of PCN-224, PCN-224(Zn), and PCNZnCy. **(E)** HAADF-STEM image and corresponding energy-dispersive X-ray spectroscopy (EDS) elemental maps of PCNZnCy. Scale bars: 100 nm. **(F)** XPS spectrum analysis of PCNZnCy, showing high-resolution spectra of **(G)** C 1s, **(H)** O 1s, **(I)** Zr 3d, and **(J)** Zn 2p core levels. **(K)** UV–Vis absorption spectra of Chrysin, PCN-224, PCN-224(Zn), and PCNZnCy. **(L)** pH- and ROS-responsive release profiles of Chrysin from PCNZnCy under different conditions (pH 5.5, 6.5, 7.4, and pH 5.5 + H₂O₂). **(M, N)** Evaluation of colloidal stability, **(M)** Long-term colloidal stability of PCNZnCy monitoring via DLS in various media 7 days (n = 3) and **(N)** Photographic visual stability assessment of PCNZnCy dispersions in water, PBS, 0.9% NaCl, and DMEM+10% FBS at days 0 and 7. Data in panels L and M are presented as mean ± SD (n = 3). ***p < 0.001, ****p < 0.0001 compared with the pH 7.4 group.

To address the multifactorial pathological barriers of infected diabetic wounds—namely bacterial burden, excessive oxidative stress, and impaired tissue regeneration—we rationally engineered a multifunctional MOF-based nanoplatform by integrating the natural flavonoid Chrysin into a zinc (Zn)-doped porphyrinic metal–organic framework (PCN-224), yielding PCNZnCy. As schematically illustrated in Figure 1A, the synthesis involved the dual-metal coordination of Zr⁴⁺ clusters and Zn²⁺ ions with tetrakis(4-carboxyphenyl)porphyrin (TCPP) ligands, followed by the post-encapsulation of Chrysin. PCN-224 has the properties of providing structural stability and a ROS-responsive porphyrin activation 29. The incorporation of Zn²⁺ enhances the metal coordination, thus increasing the antibacterial and catalytic activities and providing more anchoring points for chrysin 30. Chrysin possesses well-documented antioxidant and anti-inflammatory functions 27. What is crucial is that chrysin is chemically incorporated into the dual-metal framework rather than merely mixing the substances. Consequently, a coordinated nanosystem is obtained which can control oxidative stress, inhibit bacterial growth and promote tissue repair, features which are different from those of the ordinary single-function MOF systems.

SEM and TEM analyses confirmed that PCNZnCy preserved the well-defined spherical morphology of pristine PCN-224 and PCN-224(Zn), without obvious aggregation or structural collapse (Figure 1B-C and Figure S1-S2). These observations suggest that Zn doping and Chrysin loading did not cause obvious aggregation or structural collapse at the electron-microscopy level, consistent with the preservation of the overall MOF morphology. DLS measurements further confirmed a narrow, monodisperse hydrodynamic size distribution (Figure 1D), supporting high colloidal stability under physiological conditions. Additionally, PCNZnCy exhibited a hydrodynamic diameter of 209.4 ± 6.3 nm, a low PDI of 0.176 ± 0.021, and a zeta potential of -20.8 ± 1.6 mV (Table S1), confirming its uniform size distribution and excellent colloidal stability. High-angle annular dark-field scanning TEM combined with energy-dispersive X-ray spectroscopy (EDS) elemental mapping further verified the homogeneous co-distribution of Zr, Zn, C, N, and O across the entire nanostructure (Figure 1E and Figure S3), suggesting that Zn was distributed throughout the PCN-224 framework without evidence of large-scale segregated domains at the resolution limit of EDS mapping. XPS survey spectra confirmed the presence of all constituent elements in PCNZnCy (Figure 1F). High-resolution spectra provided detailed chemical state information: the C 1s spectrum displayed characteristic peaks at 284.80 eV (C–C/C=C), 286.71 eV (C–N), and 288.78 eV (O–C=O), corresponding to the porphyrinic ligands and Chrysin molecules (Figure 1G); the O 1s spectrum (Figure 1H) revealed multiple components associated with metal–oxygen coordination and carboxylate/phenolic oxygen species; the Zr 3d doublet at 182.92 and 185.40 eV confirmed the stable Zr⁴⁺ oxidation state within the framework; and the Zn 2p peaks at 1022.14 and 1045.13 eV clearly validated the successful incorporation of Zn²⁺ into PCNZnCy (Figure 1I-J), consistent with previously reported PCN-224-derived systems 14,21.

To probe the host–guest interplay, we explored shifts in binding energy following Chrysin loading. The subtle yet reproducible changes in the XPS spectra (Figure S4) point to electronic communication between the flavonoid and the porphyrinic scaffold, implying integral incorporation within the PCNZnCy matrix rather than simple physical adsorption. This electronic coupling might be the reason for the redox-sensitive property of the hybrid, which is important for its therapeutic effect. Concerning the optical properties, the UV–vis absorption curve of PCNZnCy (Figure 1K) presents a combined spectrum, composed of the specific Soret and Q bands of the Zn-metalloporphyrin linker and the inherent π–π* transition of the payload, suggesting a good embedding in the framework. By using UV–vis spectroscopy, the encapsulation efficiency (EE) and drug loading content (DLC) of Chrysin were determined to be 20.77% and 17.34% (w/w), respectively, confirming successful loading of this flavonoid into the framework. For establishing a reliable biological basis, two control groups were prepared: free Chrysin given at an amount equivalent to its 17.34% loading in PCNZnCy, and the empty PCN-224(Zn) carrier with Zn²⁺ concentration exactly corresponding to that detected in the drug-loaded composite by ICP-MS (Figure S5A).

The release behavior of PCNZnCy is influenced by both the changes of pH and high concentrations of ROS in the infected diabetic wound microenvironment. From Figure 1L, it can be seen that under typical physiological condition (pH 7.4), Chrysin does not release much from the matrix within 24 hours, which implies its good structural stability and low early leakage. However, under acidic buffer solutions (pH 6.5 and 5.5) or oxidative stress conditions, an obvious increase in Chrysin release is observed, and the total amount reaches over 80%. The ICP-MS results also support this, indicating the highest concentration of zinc ions at pH 5.5 together with H₂O₂ (Figure S5A). Similarly, zirconium ions show the same trend (Figure S5B), suggesting that the MOF structure decomposes rapidly in acidic and peroxide-rich conditions in infected wounds. To study the therapeutic effects of such release pattern, we have investigated the kinetic profiles of zinc ions and zirconium ions in our system. By comparing the release of zinc ions and zirconium ions in our system with that in PCN-224@ZIF-8, it is found that the addition of these components in PCN-224 delays the rapid release of zinc ions, thereby reducing the systemic toxicity. According to the mechanism, this dual response is consistent with the known degradation chemistry of porphyrin MOFs, where the protonation of the bridging carboxylates and oxidation of the organic linkers by ROS damage the crystal network 14,21. In conclusion, these findings establish PCNZnCy as an infection-adaptive nanoplatform that couples bactericidal Zn²⁺ and antioxidant Chrysin delivery at sites where acidosis and oxidative stress converge.

To ensure that the accelerated release arises from specific triggers rather than from nonspecific breakdown, we investigated the colloidal stability of PCNZnCy in several physiologically relevant fluids (Figure 1M–N). During a period of seven days, the DLS technique indicated that the nanoparticles preserved a constant hydrodynamic diameter in water, saline, PBS and the medium supplemented with serum (DMEM containing 10% FBS), without any aggregation or settling. The significant stability under neutral and protein-rich conditions is in sharp contrast to the quick drug efflux observed in acidic or oxidative environments, which supports the idea that the liberation of chrysin is driven by stimulants. We acknowledge, however, that DLS cannot fully detect very subtle structural changes; higher-resolution imaging or scattering methods would be needed to completely exclude such possibilities. Taken together, these findings position PCNZnCy as an integrated, environment-responsive platform with verified colloidal dispersion, distinct from conventional MOF carriers, and provide a physicochemical rationale for its combined antibacterial, antioxidant, and regenerative actions in infected diabetic wounds.

### Synergistic antibacterial and anti-biofilm performance of PCNZnCy against MRSA and *P. aeruginosa*

**Figure 2:**
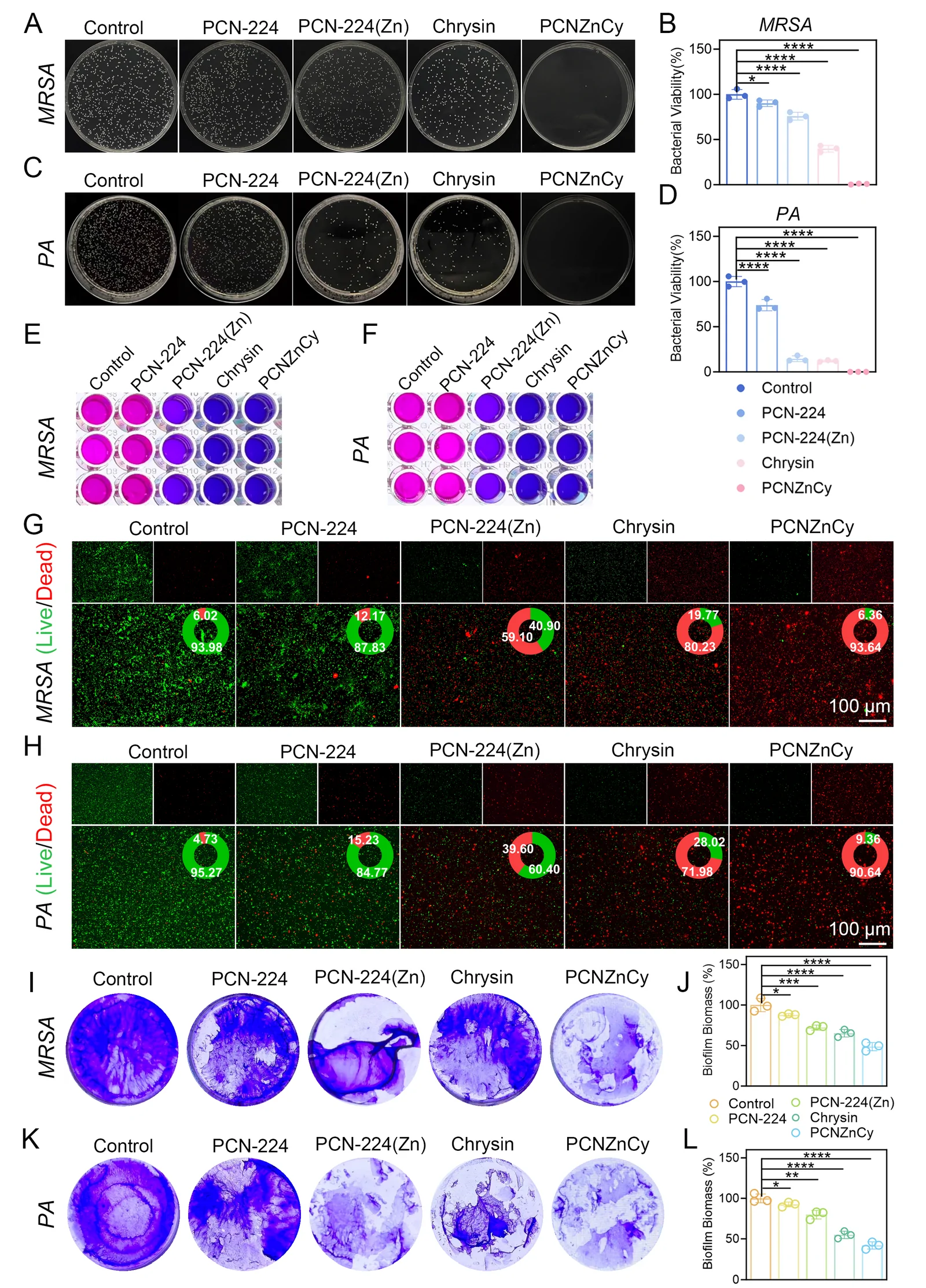
** Antibacterial and anti-biofilm performance of PCNZnCy nanoplatform. (A, C)** Representative Photographs of colony-forming unit (CFU) assay for **(A)** MRSA and **(C)** PA after treatment with Control, PCN-224, PCN-224(Zn), Chrysin, and PCNZnCy.** (B, D)** Quantification of bacterial viability using the CFU assay**. (E, F)** Resazurin metabolic assay of MRSA and PA after different treatments.** (G, H)** Live/Dead fluorescence staining of MRSA and PA (green: live; red: dead). Inset pie charts denote quantitative analysis. Scale bars: 100 μm. **(I, K)** Crystal violet staining of MRSA and PA biofilms after different treatments.** (J, L)** Quantification of biofilm biomass. Data are mean ± SD (n = 3); ns, not significant; *p < 0.05, **p < 0.01, ***p < 0.001, ****p < 0.0001.

The antibacterial performance of PCNZnCy was first evaluated against two clinically relevant, highly drug-resistant pathogens, MRSA (Gram-positive) and PA (Gram-negative), which are major causes of chronic infected wounds. As shown in Figure 2A–D, PCNZnCy exhibited nearly complete eradication of both planktonic MRSA and PA, whereas PCN-224(Zn) and free Chrysin displayed moderate antibacterial effects compared with control and PCN-224. This demonstrates that the Zn²⁺ ions in PCN-224(Zn) further confer antibacterial activity, and that the incorporation of both PCN-224(Zn) and free Chrysin in PCNZnCy supports its superior antibacterial effect. Quantitative colony-forming unit counting revealed over 99% drop in viable bacterial counts for both MRSA and PA (Figure 2B, D), indicating that the PCNZnCy formulation exerts a synergistic killing effect that surpasses what each component can achieve alone. The MIC and MBC values for PCNZnCy were 64/128 μg mL⁻¹ against MRSA and 32/64 μg mL⁻¹against PA (Table S2). These thresholds place PCNZnCy firmly in the potent bactericidal category against both MDR strains. Notably, the lower values against the Gram-negative isolate suggest slightly greater susceptibility. Altogether, these quantitative readouts offer robust efficacy benchmarks and should guide dose selection for subsequent preclinical and translational work.

To get further confirmation about the inhibition of bacterial metabolism, we did resazurin reduction tests. In the PCNZnCy treated groups, the metabolic activity obviously decreased (Figure 2E-F), which was consistent with the decline in the number of colony forming units (CFU). In order to observe the condition of the cells more clearly, fluorescence-based live/dead staining was performed (Figure 2G-H). After treating with PCNZnCy, most of the pathogenic bacteria exhibited a strong red fluorescence, indicating membrane damage and irreversible injury. In contrast, the control samples and those treated with other components showed mainly green fluorescence, suggesting healthy and living cells. Quantitatively, this treatment made more than 90% of both MRSA and PA ineffective. Remarkably, the antibacterial efficiency of PCNZnCy is much higher than that of the previously reported PCN-224 based analogues, such as PCN-224@ZIF-8 and Van-PCN-224 19,21. Several possible mechanisms might explain this improvement. It is considered that the zinc ions released from the MOF backbone could destroy bacterial membranes, cause oxidative damage and disrupt the intracellular redox balance 14. Additionally, the flavonoid chrysin is thought to interfere with the energy metabolism and inhibit the synthesis of nucleic acids 26. Moreover, the PCN-224(Zn) framework, due to its Zr⁴⁺/Zn²⁺ coordination and the TCPP derived porphyrin linkers, may promote electron transfer and generate ROS inside 31. Importantly, unlike the conventional photosensitizing MOFs which require external light stimulation 31-33, PCNZnCy has an auto-sustained redox-metal-organic cooperative killing pathway to achieve powerful bactericidal effect without external energy. Furthermore, the zinc ions from biological sources show significant antibacterial effects and possess much better biocompatibility than heavy metals like Ag⁺, Cd²⁺ and Hg²⁺. Although the combined mechanisms and their agreement with the observed effectiveness are reasonable, we acknowledge a limitation. The present data cannot precisely distinguish the contributions of each component. Further research is needed to clarify these fractional influences.

Bacterial biofilms are responsible for the recurrence of chronic infections and often affect the treatment results 34,35, so we studied the anti-biofilm activity of PCNZnCy systematically. By using crystal violet staining, we found that PCNZnCy could greatly destroy the formed MRSA and PA biofilms (Figure 2I–L), decreasing the total amount by about 60% - which is much more significant than that caused by PCN-224, PCN-224(Zn) or chrysin alone, indicating its better efficacy. It is obvious that PCNZnCy not only inhibits the planktonic growth but also actively destroys the extracellular polymeric substance (EPS) that usually protects the pathogens from both antibiotics and host immunity. In order to confirm these structural changes, we measured the extracellular polysaccharides, the main component of EPS. The levels of polysaccharides in the PCNZnCy treated groups were reduced about 30% compared with the untreated controls for both types of bacteria (Figure S6), which confirms that the substance attacks the matrix framework. In summary, these findings show that PCNZnCy weakens the mature biofilm structure by decomposing its specific polysaccharide backbone, a mechanism that may explain its higher disassembling efficiency.

Mechanistically, the antibiofilm activity hinges on the nanoconfined co-delivery system uniquely enabled by the Zn-doped PCN-224 framework. By sequestering both Zn²⁺ and hydrophobic Chrysin within the same MOF cavity, our design ensures their synchronized release in close spatiotemporal proximity. When exposed to the acidic, ROS-rich conditions typical of infected tissues, PCNZnCy destabilizes predictably, triggering a concentrated surge of both agents (Zn²⁺ and Chrysin). The simultaneous release has several advantages: it breaks down the extracellular polymeric matrix, damages the bacterial membrane directly and inhibits the basic metabolic processes. This comprehensive attack can overcome the usual efflux and quiescence strategies which protect the bacteria in the biofilm. Compared with single functional methods, the adaptability and release of multiple substances show a remarkable improvement. Our findings show that PCNZnCy acts as a real non-antibiotic platform, exhibiting high efficiency for both MRSA and *P. aeruginosa*, and able to eliminate planktonic cells and destroy mature biofilms. The substantial increase in its efficiency in comparison with the individual components indicates the cooperation among Zn²⁺, chrysin and the MOF skeleton, although the exact synergistic mechanism still needs further investigation. PCNZnCy offers an effective, non-antibiotic antibacterial strategy for MDR and biofilm-associated infections. However, whether repeated exposure to PCNZnCy induces bacterial resistance warrants further investigation using serial-passage resistance-induction assays.

### PCNZnCy exhibits potently enhanced free radical scavenging activity

**Figure 3:**
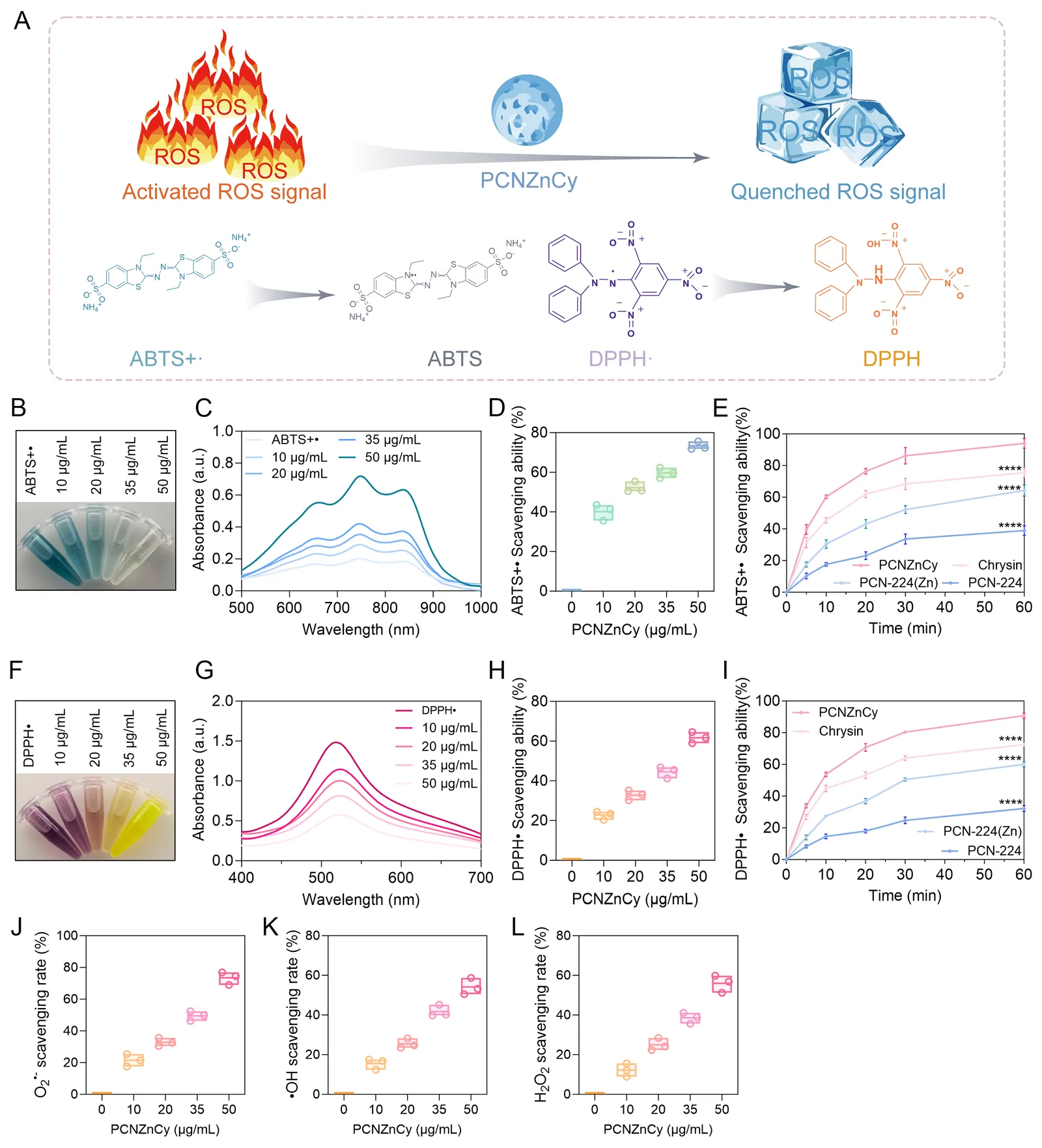
** Antioxidant performance of PCNZnCy. (A)** Schematic illustration of the free radical scavenging mechanism of PCNZnCy. **(B–E)** ABTS⁺• radical scavenging ability assay. **(B)** Photographs of the fading coloration of ABTS⁺• solution with increasing PCNZnCy concentration, **(C)** corresponding UV–vis absorption spectra of ABTS⁺•, and **(D)** Quantitative, concentration-dependent scavenging efficiency after treatment with different concentrations of PCNZnCy. **(E)** Time-dependent ABTS⁺• scavenging efficiency of PCNZnCy, chrysin, PCN-224(Zn), and PCN-224. **(F–G)** DPPH• radical scavenging assay. **(F)** Photographs of the fading coloration of DPPH• solution with increasing PCNZnCy concentrations, **(G)** corresponding UV–vis absorption spectra of DPPH• after PCNZnCy treatment, and **(H)** Quantitative, concentration-dependent DPPH• scavenging ratios. **(I)** Time-dependent DPPH• scavenging kinetics of PCNZnCy, chrysin, PCN-224(Zn), and PCN-224. **(J-L)** Concentration-dependent scavenging efficiency of O₂·⁻, ·OH, and H₂O₂ after treatment with different concentrations of PCNZnCy. Data are mean ± SD (n = 3); ns, not significant; *p < 0.05, **p < 0.01, ***p < 0.001, ****p < 0.0001.

Recently, the intrinsic antioxidant activity of MOFs has attracted increasing attention for therapeutic applications 12,16. Completion of infected diabetic wounds depends on not only the elimination of bacteria but also the reduction of excessive harmful ROS which cause local inflammation, inhibit the functions of the local cells and accelerate matrix destruction. In order to examine this antioxidant effect, we used the common ABTS⁺• and DPPH• assays (Figure 3A). The addition of PCNZnCy to the ABTS⁺• working solution makes it lighten its deep blue-green color quickly, and the degree of decolorization is closely related to the amount added (Figure 3B). UV–vis spectrophotometry verified these visual results and found that the absorbance at 734 nm decreased continuously with the increase of PCNZnCy concentration (Figure 3C). The spectral analysis indicated a significant dose-response relationship; when the concentration was 50 μg·mL⁻¹, PCNZnCy could suppress about 80% of the initial ABTS⁺• absorbance (Figure 3D), and it performed better than ZIF-8 15,16 and UiO-66 17 in this test. What is more important, the kinetic measurements clearly showed that PCNZnCy could neutralize the ABTS⁺• radicals with much faster kinetics and achieve a more complete end point than free Chrysin, the PCN-224(Zn) analogue, or the pure PCN-224 structure (Figure 3E). These spectroscopic data collectively affirm PCNZnCy‘s potent free-radical-quenching capability.

When the second radical probe was detected, the stable DPPH• test gave the same results. With the continuous addition of PCNZnCy, the solution turned colorless and the bleaching effect was related to the dose (Figures 3F- G). When the concentration reached 50 μg mL⁻¹, the scavenging rate was over 60% (Figure 3H). Time course curves (Figure 3I) showed that PCNZnCy could more efficiently and thoroughly quench DPPH• than Chrysin, PCN-224(Zn) and the unmodified PCN-224 framework, and its efficiency was higher than those of other antioxidant metal organic frameworks such as curcumin-zinc MOFs 36. To study whether there is any special advantage of the nanostructure apart from merely mixing, PCNZnCy was compared with its free components which contained the same amount of loading as the composite at 50 μg mL⁻¹. The assembled PCNZnCy exhibited better performance in both ABTS•⁺ and DPPH• systems than either component separately (Figures 3E, I), indicating a synergistic effect on radical quenching rather than a simple additive effect. Since hydroxyl radicals (•OH), superoxide anion radical (O₂•⁻) and hydrogen peroxide (H₂O₂) are the main causes of oxidative tissue damage 37,38, we also examined the ability of PCNZnCy to eliminate these radicals (Figures 3J-L). The nanomaterial showed a dose-dependent inhibition for all three free radicals, demonstrating its wide-spectrum ROS inhibiting ability. It should be mentioned that PCNZnCy acts as an antioxidant and does not imitate the activity of enzymes like peroxidase or oxidase. This is significant because if it generates extra •OH or O₂•⁻, it would only worsen the harmful oxidative condition in diabetic wounds. Conversely, its consumption of ROS is consistent with the therapeutic purpose of restoring the redox balance.

Mechanistically, the enhanced antioxidant ability of PCNZnCy is due to the cooperative redox reactions of its two different active sites. By itself, Chrysin eliminates radicals by means of its phenolic hydroxyl groups 39, but its application is greatly limited by low water solubility and chemical instability. Furthermore, the Zn-doped PCN-224(Zn) framework possesses redox properties, probably through electron transfer at the metalloporphyrin centers and coordination of Zn²⁺ ions. The incorporation of Chrysin into this porous structure not only enhances the accessibility of radicals to these sites but may also promote electronic coupling between the flavonoid and the framework. In various tests for radical-scavenging, this combined system shows better results than the sum of its separate parts, indicating a real synergistic effect instead of just additive effects.

The chronic wound of a diabetic patient is a special hostile micro-environment. Due to continuous inflammation, bacterial colonization and abnormal immune responses, a lot of ROS is generated which damages the neighboring cells and decreases the extracellular matrix. Under such circumstances, the broad-spectrum scavenging ability of PCNZnCy, particularly for ABTS⁺• and DPPH• radicals, is very important. It can interfere with the self-perpetuating cycle of oxidative damage and inflammatory signals, thereby preventing the healing process from being affected. When used together with its original antibacterial action, it constitutes a powerful dual-function system. The infection is controlled and the redox balance is recovered, solving the two main problems for recovery. As a result, the local environment becomes an ideal condition for re-vascularization and parenchymal regeneration, thereby enabling durable closure.

### PCNZnCy regulates cellular redox homeostasis and preserves mitochondrial function via activation of the Nrf2/HO-1 axis

**Figure 4:**
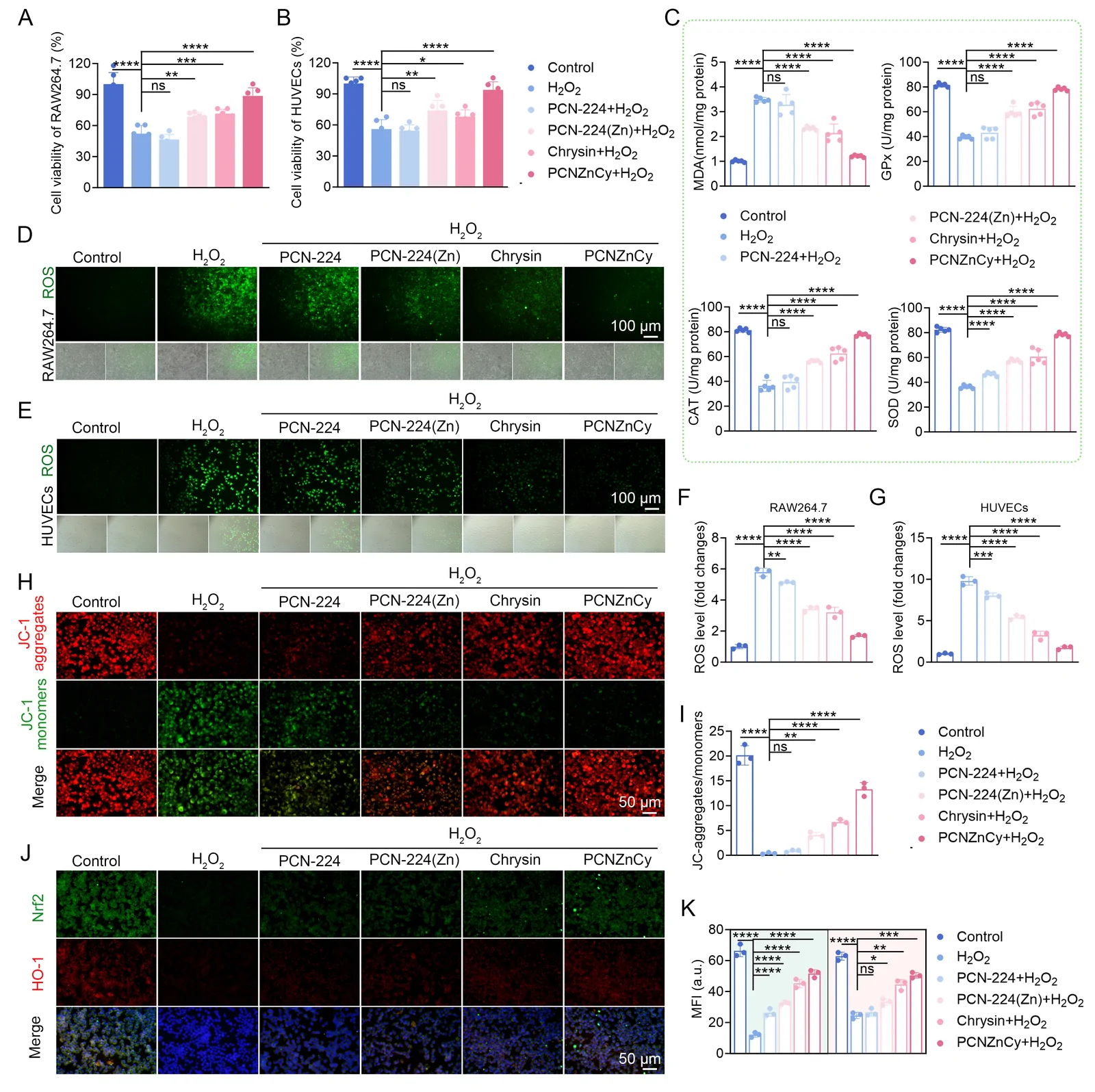
** PCNZnCy protects RAW264.7 and HUVECs cells against H₂O₂-induced oxidative stress and mitochondrial dysfunction. (A, B)** CCK-8 assay for cell viability of **(A)** RAW264.7 macrophages and **(B)** HUVECs with different treatments. **(C)** Quantitative analysis of MDA, GPx, CAT, and SOD in RAW264.7 cells. **(D-G)** Fluorescence microscopy images of DCFH-DA-stained **(D)** RAW264.7 cells and **(E)** HUVECs (scale bar: 50 μm), with corresponding quantitative analysis of ROS fluorescence intensity in **(F)** RAW264.7 and **(G)** HUVECs. **(H, I)** mitochondrial membrane potential (ΔΨm) evaluation via **(H)** JC-1 staining assay (scale bar: 50 μm), and **(I)** corresponding quantification of the aggregate/monomer ratio (ΔΨm). Red: JC-1 aggregates, healthy mitochondria; green: JC-1 monomers, depolarized mitochondria. **(J)** immunofluorescence staining (scale bar: 50 μm) and **(K)** quantitative analysis of Nrf2 (green) and HO-1 (red) expression in RAW264.7 cells, with DAPI (blue) for nuclei. Data are mean ± SD (n = 3); ns, not significant; *p < 0.05, **p < 0.01, ***p < 0.001, ****p < 0.0001.

To elucidate the cytoprotective and pro-regenerative potential of PCNZnCy at the cellular level, we systematically evaluated its ability to protect RAW264.7 macrophages and human umbilical vein endothelial cells (HUVECs) from oxidative damage induced by H₂O₂, a model that closely mimics the excessive oxidative stress microenvironment of infected diabetic wounds. A concentration of 200 μM H₂O₂ was chosen for oxidative stress investigation, as it consistently reduced cell viability, representing a clinically relevant sublethal oxidative injury level that allows evaluation of cytoprotective and regenerative effects 14. Exposure to 200 μM H₂O₂ greatly reduced the viability of both cell types (Figure 4A-B). Free Chrysin or PCN-224(Zn) individually provided certain degree of protection, but the complete PCNZnCy formulation could nearly restore the cell survival to the normal level. This significant improvement may be due to a cooperative action in the hybrid structure, which is better than the expected result of adding the separate components together. The cytoprotective property was also reflected in the biochemical changes.

In RAW264.7 macrophages after H₂O₂ treatment, MDA concentrations increased whereas the activities of GPx, CAT and SOD decreased. The administration of PCNZnCy could markedly decrease MDA levels and recover the activities of these three enzymes to nearly normal values (Figure 4C), suggesting a complete restoration of the cellular redox state. Direct visualization of intracellular ROS using the DCFH-DA probe further confirmed the exceptional ROS-scavenging capacity of PCNZnCy in both RAW264.7 macrophages (Figure 4D, F) and HUVECs (Figure 4E, G). Compared with free Chrysin, PCN-224, and PCN-224(Zn), PCNZnCy exhibited the greatest suppression of ROS fluorescence intensity, indicating superior intracellular redox-regulatory efficiency. In agreement with previous reports on redox-active MOFs 12,16, the observed antioxidant effect may involve direct radical neutralization by phenolic hydroxyl groups of Chrysin, together with electron donation from the porphyrinic framework. Significantly, Zn-doped porphyrinic frameworks not only facilitate cellular uptake and controlled intracellular delivery of Chrysin but also act as intrinsic redox mediators, jointly amplifying the antioxidant response.

Because mitochondrial dysfunction is a critical downstream consequence of uncontrolled oxidative stress and a key determinant of cell fate during chronic wound pathology 40,41, subsequently, mitochondrial membrane potential (ΔΨm) was assessed using JC-1 staining. Exposure to H₂O₂ caused a significant drop in ΔΨm (Figures 4H-I) by the conversion of red JC-1 aggregates to green monomers (depolarized mitochondria). In contrast, the red/green ratio of PCNZnCy-treated cells is nearly the same as that of the untreated group, suggesting the effective preservation of mitochondrial polarization. It is important to keep ΔΨm not only for maintaining ATP production and preventing cytochrome c efflux but also for keeping the functional ability of repair cells during the wound healing process. Therefore, by reducing the cytosolic ROS level, PCNZnCy seems to break the destructive ROS-mitochondria-cell death cycle, thus maintaining the cell's energy metabolism. To investigate the underlying redox mechanism further, we studied the Nrf2/HO-1 pathway, which is a recognized hub for antioxidant and cytoprotective signals 42,43. The immunofluorescence staining of RAW264.7 cells showed that PCNZnCy treatment could increase the nuclear import of Nrf2 and the expression of HO-1 (Figure 4J-K), indicating a coordinated transcriptional response which strengthens its protective effect. Compared with single-component treatments, the PCNZnCy group showed the strongest activation of Nrf2 and induction of HO-1, indicating that the nanoplatform can effectively enhance the endogenous antioxidant signal. Western blot analysis also confirmed these results, demonstrating that PCNZnCy could significantly restore the protein expression of Nrf2 and HO-1 suppressed by H₂O₂, better than PCN-224, PCN-224(Zn) and free Chrysin (Figure S7). This provides further causal evidence by functional validation of the activation of Nrf2/HO-1, which is necessary for redox protection and healing benefits.

Mechanistically, a supporting study indicates that chrysin affects the ERK/Nrf2 pathway in glioblastoma cells, reinforcing its role in redox regulation 44. It might be possible that chrysin from PCNZnCy binds to Keap1 and interferes with its binding to Nrf2, thus preventing the degradation of Nrf2. As a result, Nrf2 enters the nucleus and activates the downstream antioxidant response elements (AREs), such as HO-1, which promotes the production of antioxidant substances like SOD and GPx, thereby improving the cell's resistance to oxidative stress 45. The redox active Zn-porphyrin centers in PCNZnCy can produce and release signals according to ROS, which also activates Nrf2. Considering that HO-1, an antioxidant 46, anti-inflammatory and pro-angiogenic enzyme, is very important, it provides a reasonable explanation for the observed protective effects. Although our results show the involvement of this pathway, they are not enough to confirm the definite mechanism. Besides its passive scavenging of oxidants, PCNZnCy regulates the redox state actively by inhibiting the release of oxidants, maintaining the membrane integrity of mitochondria and restoring the endogenous Nrf2/HO-1 axis. This multiple level regulation is advantageous for the immune homeostasis and vascular regeneration in diabetic wounds. Therefore, our *in vivo* findings associate the redox changes with tissue repair, indicating that the efficiency of PCNZnCy depends on its comprehensive biological activities rather than its single antioxidant functions.

### PCNZnCy enhances endothelial cell function and angiogenic potential under oxidative stress

**Figure 5:**
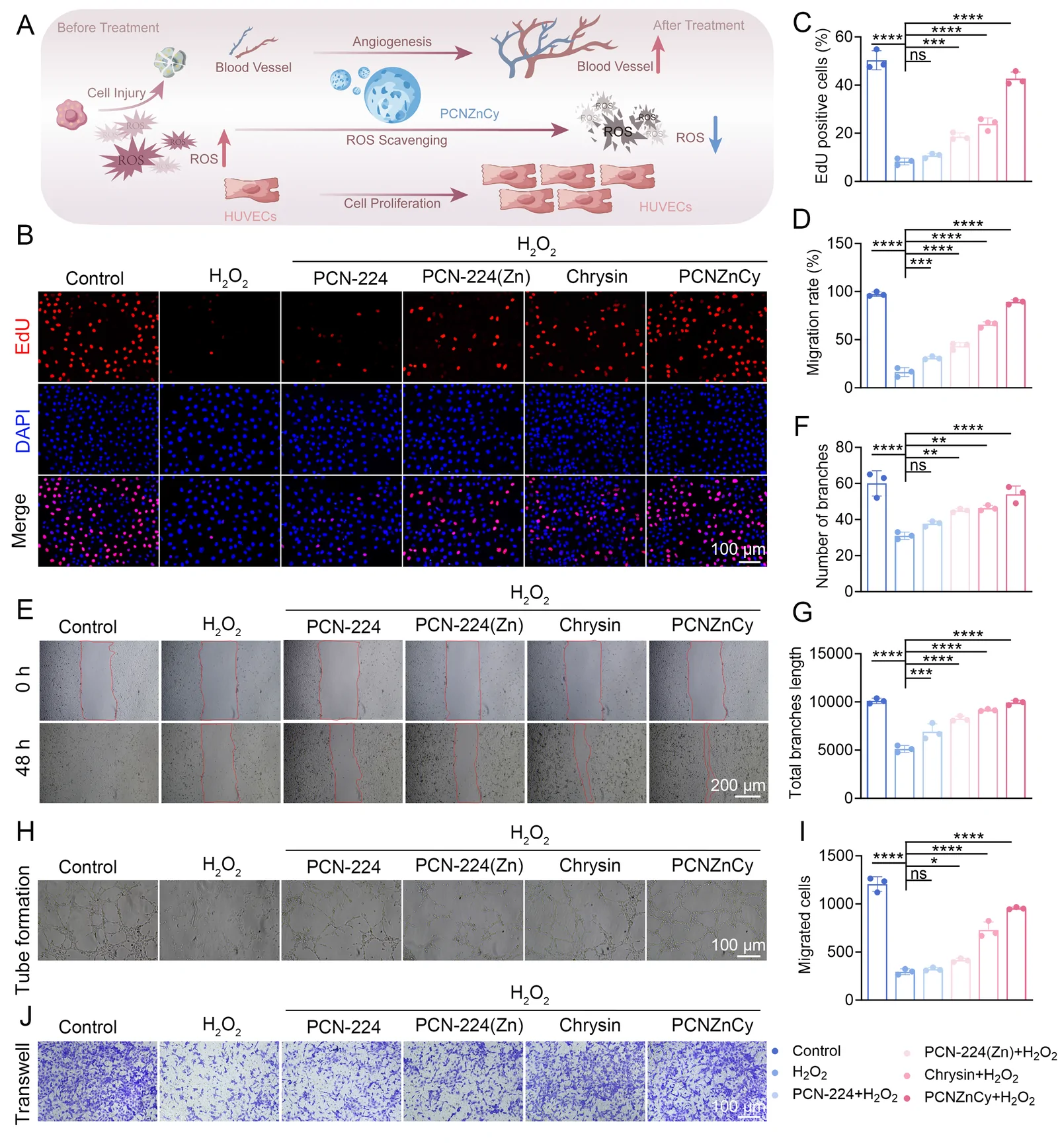
** PCNZnCy promotes HUVEC proliferation, migration, and angiogenesis under oxidative stress**. **(A)** Schematic diagram illustrating the protective mechanism of PCNZnCy on HUVEC cells under oxidative stress. **(B)** EdU staining and **(C)** quantification of HUVECs proliferation treated with H₂O₂ and different formulations. Red, proliferating cells; blue, DAPI-stained nuclei. Scale bar: 100 μm. **(D, E)** Representative images of a scratch assay and quantification of the migration rate at 0 h and 48 h. Scale bar: 200 μm. **(F-H)** Angiogenic capacity assay, showing **(F)** number of branches, with quantification of **(G)** total tube length and **(H)** representative tube formation images on Matrigel. Scale bar: 100 μm. **(I, J)** Quantification of migrated cells in each group and transwell assay images. Scale bar: 100 μm. Data are mean ± SD (n = 3); ns, not significant; *p < 0.05, **p < 0.01, ***p < 0.001, ****p < 0.0001.

Successful tissue regeneration critically depends on angiogenesis, a process orchestrated by endothelial cells whose proliferative and migratory functions are severely impaired in the oxidative microenvironment of chronic wounds 2,47. We therefore investigated whether PCNZnCy could directly promote endothelial repair and angiogenic activity under H₂O₂-induced oxidative stress (Figure 5A). EdU staining showed that oxidative stress significantly curtailed HUVEC proliferation, with the fraction of EdU-positive cells plummeting under ROS exposure (Figure 5B–C). PCNZnCy treatment not only rescued this activity but also outperformed free Chrysin and PCN-224(Zn) in restoring endothelial growth. This finding implies that our nanoplatform effectively overcomes oxidative stress-induced cell-cycle arrest, thereby restoring the proliferative potential essential for vascular repair. Regarding motility, PCNZnCy significantly promoted the migration of endothelial cells, which is essential for angiogenic sprouting. In the scratch wound closure (Figure 5D-E) and transwell migration assays (Figure 5I-J), the group treated with PCNZnCy showed the quickest closure rates and the most distinct directional movement under oxidative stress. In all the comparisons, this dual-functional substance always performed better than single treatments, indicating its excellent ability to completely recover the motility of endothelial cells damaged by ROS.

When assessing the restoration of functions, PCNZnCy can restore the ability of endothelial cells to arrange themselves. In the usual experimental matrigel experiments, the HUVECs treated with H₂O₂ only formed broken and discontinuous traces, which are usually indicative of angiogenic failure. But after the application of PCNZnCy, extensive and well-connected capillary networks were constructed, with a considerable increase in the total tube length and the number of branch points (Figure 5F-H). Normally, oxidative damage affects angiogenesis by disturbing the cytoskeleton and blocking the promoting effect of migration. By reducing the intracellular ROS (Figure 4) and restoring the redox balance, PCNZnCy overcomes these obstacles and promotes endothelial proliferation, migration and tubulogenesis. This approach is essentially different from the conventional pro-angiogenic methods which rely on the provision of external growth factors. Instead of giving external signals, PCNZnCy alters the local microenvironment; it eliminates the oxidative barriers, so that the endogenous regenerative mechanism of endothelium works automatically and efficiently.

Our results indicate that PCNZnCy can overcome an important problem in the treatment of chronic wounds, particularly the inability of vascular regeneration under continuous oxidative stress. It maintains the function of endothelium in this difficult condition, which promotes the establishment of a vascular network with blood flow, guaranteeing sufficient oxygen and nutritional supply as well as immune monitoring at the wound area. As a result, it facilitates effective tissue reconstruction and repair. Rather than simply fighting infection or scavenging free radicals, PCNZnCy appears to rebuild the structural framework needed for healing to proceed. The current *in vitro* data using HUVECs show clear pro-angiogenic activity. While the current evidence demonstrates effective angiogenic effects in HUVECs, future investigations incorporating additional cell types will further validate PCNZnCy's wound-healing-promoting effects.

### PCNZnCy enhances infection clearance and tissue regeneration PA–infected diabetic wounds

**Figure 6:**
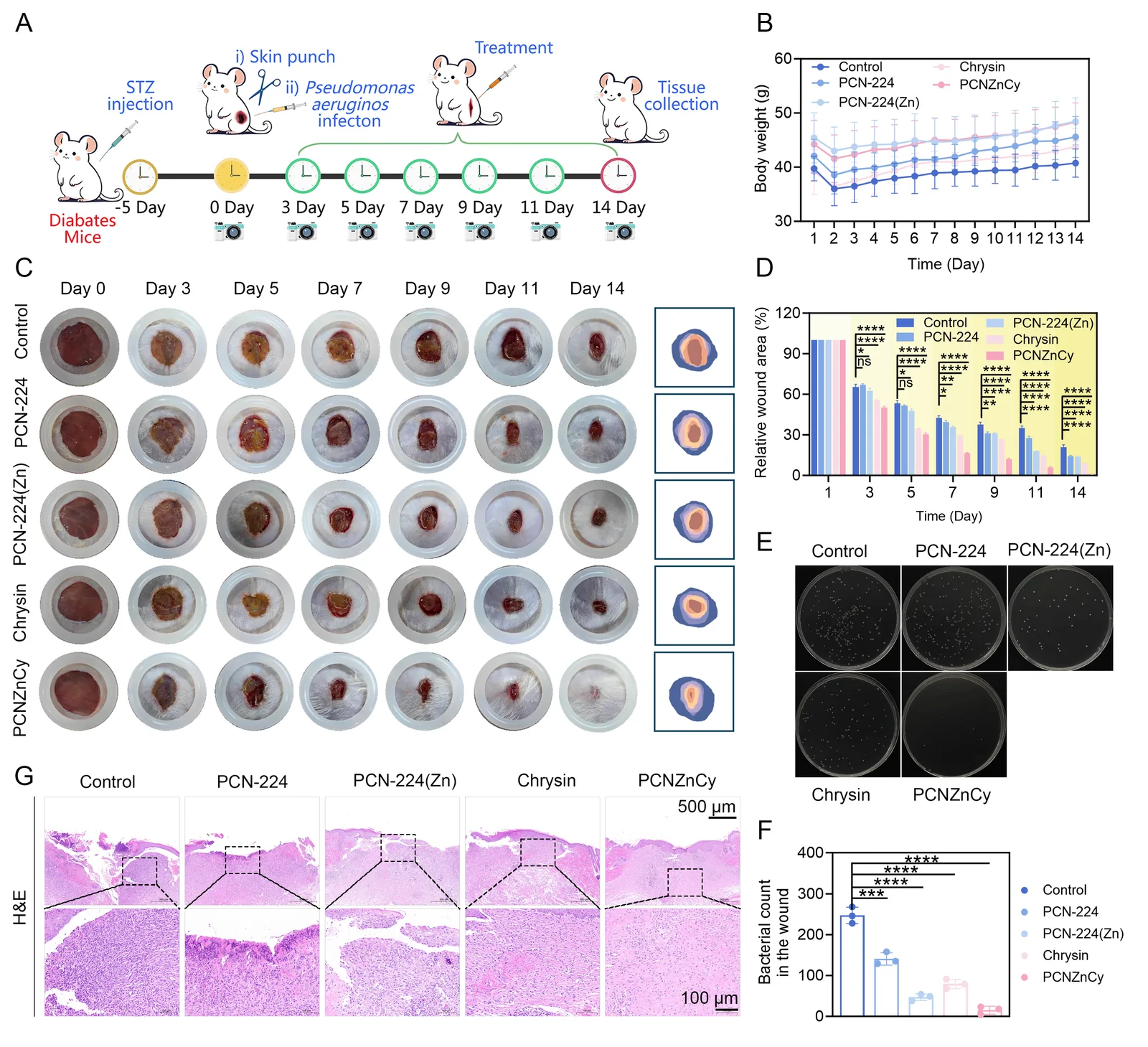
** Therapeutic efficacy of PCNZnCy in PA–infected diabetic wounds. (A)** Schematic diagram of the experimental timeline, showing streptozotocin (STZ)-induced diabetes, wound creation and *PA* infection, treatment regimen, and sample collection. **(B)** Body weight monitoring of diabetic mice over a 14-day treatment. **(C)** Representative photographs of wound closure from Day 0 to 14 after different treatments and corresponding ImageJ analysis of wound closure kinetics. **(D)** Quantification analysis of the relative wound area over time. **(E)** Representative photographs of the CFU assay from infected wound tissues, and corresponding **(F)** quantification of CFU counts. **(G)** H&E staining of wound tissues after different treatments on day 14. Scale bar: 100 μm. Data are mean ± SD (n = 6 mice per group); ns, not significant; *p < 0.05, **p < 0.01, ***p < 0.001, ****p < 0.0001.

Diabetic wounds represent a convergence of multiple pathological barriers, including impaired immunity, persistent bacterial infection, chronic inflammation, and excessive oxidative stress under hyperglycemic conditions, all of which synergistically hinder effective tissue repair 14,31. To reproduce the clinical problems commonly met in real situations, we tested the effect of PCNZnCy in a diabetic wound model with PA infection, which is a common and hard-to-treat pathogen often found in non-healing wounds (Figure 6A). It was applied topically at a concentration of 100 µg per wound (a 2 mg/mL sterile PBS solution of 50 µL), beginning on day 0 and being applied every 24 hours until the end of the 14-day period. During this treatment, the diabetic mice kept their body weight unchanged and did not exhibit any obvious local irritation or systemic discomfort, suggesting good biocompatibility and systemic tolerance (Figure 6B). In order to confirm the continuous high blood sugar condition, the fasting blood glucose levels were measured; all the groups had glucose levels above 16.7 mmol/L from the fifth day after STZ injection, and this increase lasted throughout the 14-day observation period (Fig. S8). Altogether, the significant safety features shown under strict diabetic conditions provide preliminary evidence for the practical application of PCNZnCy in treating stubborn chronic wounds.

Macroscopic examination and quantitative measurements of wound area clearly revealed a distinct therapeutic edge for PCNZnCy over the control regimens. In contrast to the saline control and the groups given PCN-224, PCN-224(Zn) or free Chrysin individually, none of which resulted in wound closure until the 14th day, the wounds treated with PCNZnCy showed significantly accelerated contraction and good re-epithelialization, approaching complete closure by the 14th day (Figure 6C-D). Therefore, these quantitative results suggest that PCNZnCy can promote faster wound healing especially in diabetic patients with active bacterial infection. Particularly, this faster closure is closely related to the inhibition of infection. The amount of residual bacteria in the wound tissues was also measured to support this, showing that PCNZnCy could reduce the bacterial load of PA more than 95% compared with other treatment groups (Figure 6E-F). This elimination indicates the strong *in vivo* antibacterial activity and infection resolution of PCNZnCy.

Histological assessment gave a clearer picture of how the tissue actually remodeled beneath the surface. Hematoxylin and eosin staining shows that wounds healed with saline or single agents contain a lot of inflammatory cells, disordered granulation tissue and incomplete epidermal regeneration. However, wounds treated with PCNZnCy have organized granulation tissue, less inflammatory infiltration and a fully covered epidermis (Figure 6G). Such morphological features indicate a normal and rapid repair process, indicating that PCNZnCy can enhance both the speed and quality of skin regeneration. From the viewpoint of quantitative analysis, the tissue homogenate results prove that PCNZnCy remarkably reduces the amounts of pro-inflammatory cytokines (TNF-α, IL-1β, IL-6) and related mediators (iNOS, CXCL1, IFN-γ) compared with the control group. At the same time, it increases the expressions of some repair-related markers like IL-10, ARG1 and TGF-β, thereby altering the local immune condition to a repairing one (Figure S9). The good performance of PCNZnCy is mainly due to its Zn-doped MOF structure which acts as a co-delivery carrier for Zn²⁺ ions, an antibacterial substance and the flavonoid Chrysin with antioxidant and anti-inflammatory properties. Besides, the release behaviour of the system is regulated by pH and ROS levels (Figure 1N). In the acidic and oxidized environment of infected diabetic wounds, this design ensures that the therapeutic components are released at the specific place, improving the local efficiency and decreasing the systemic adverse effects and off-target toxicity obviously. Such microenvironment-adaptive therapeutic strategies align with emerging approaches that integrate smart material design with disease-responsive drug release to address complex pathological settings 48,49.

Importantly, these results, consistent with *in vitro* data, showed that PCNZnCy exhibits strong antibacterial and antibiofilm activity against MRSA and PA (Figure 2). We consistently anticipated that PCNZnCy would perform similarly to or better in the polymicrobial model, given its dual-metal synergy targeting both Gram-positive and Gram-negative bacteria. Collectively, these *in vivo* results demonstrate that PCNZnCy effectively addresses PA-infected diabetic wounds by simultaneously suppressing bacterial infection, alleviating inflammatory and oxidative stress, and promoting high-quality tissue regeneration. While this validation was performed specifically for PA-infected diabetic wounds, the *in vitro* broad-spectrum activity suggests potential applicability to other clinically relevant pathogens and polymicrobial models. Collectively, these *in vivo* results demonstrate that PCNZnCy effectively addresses multiple pathological obstacles in diabetic-infected wounds by simultaneously suppressing bacterial infection, alleviating inflammation and oxidative stress, and promoting high-quality tissue regeneration. This multifunctional nanoplatform therefore represents a promising therapeutic strategy for treating chronic, infection-complicated diabetic wounds. Future work will evaluate PCNZnCy in MRSA/PA co-infected diabetic wounds to establish its efficacy under more complex microbiological conditions.

### PCNZnCy promotes angiogenesis and collagen remodeling through activation of Nrf2/HO-1 signaling in diabetic infected wounds

**Figure 7:**
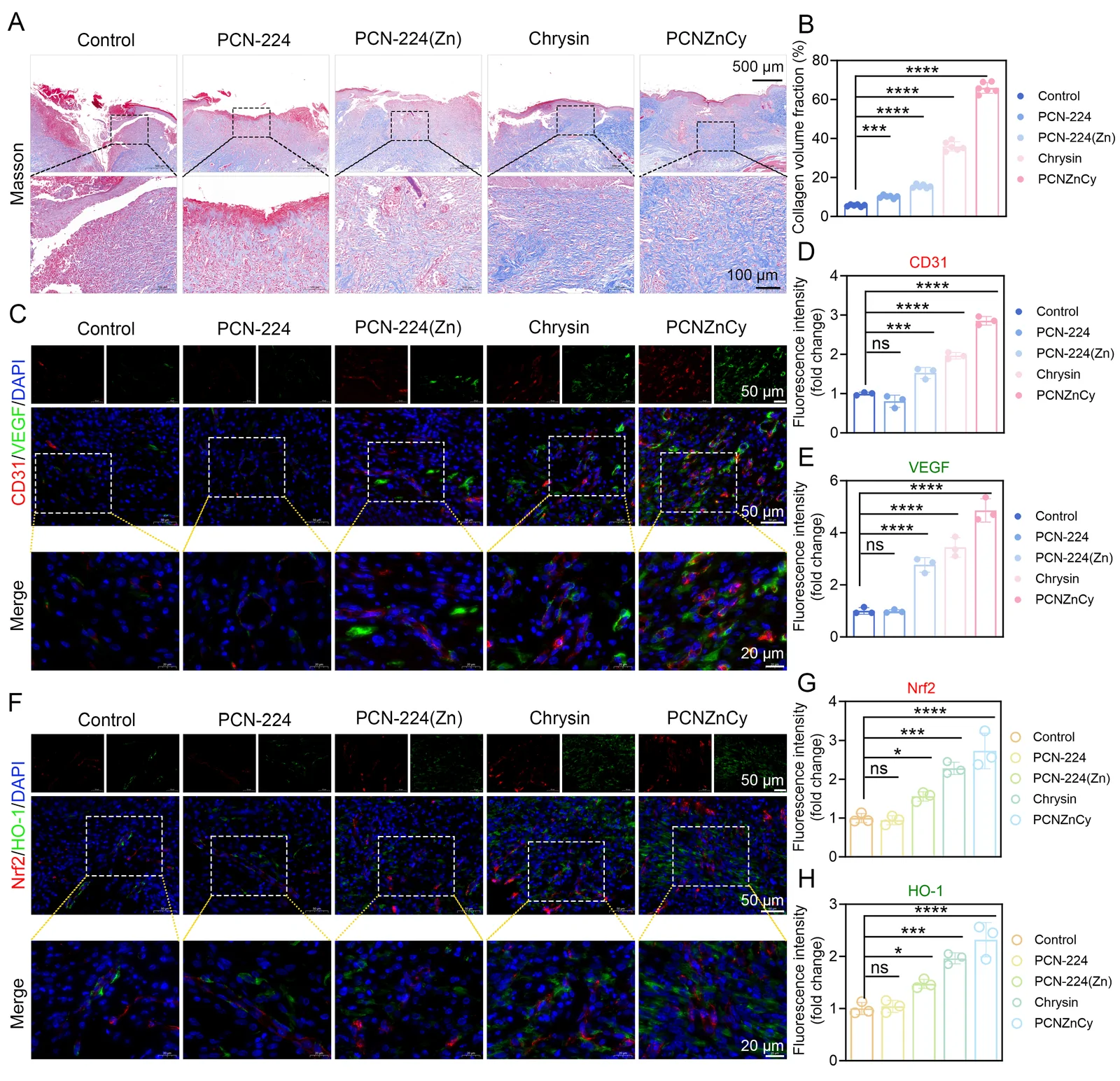
** PCNZnCy enhances angiogenesis and activates antioxidant signaling in diabetic infected wounds. (A)** Masson’s trichrome staining of wound sections on day 14. Scale bar: 100 μm.** (B)** Quantification analysis of collagen volume fraction. **(C)** Immunofluorescence co-staining of CD31 (red) and VEGF (green) in wound tissues with DAPI in blue (Nuclei counterstained). Scale bar: 20 μm. **(D, E)** Quantification of the mean fluorescence intensity for CD31 and VEGF. **(F)** immunofluorescence staining for Nrf2 (red) and HO-1 (green) with DAPI counterstaining in wound sections. Scale bar: 20 μm.** (G, H)** Quantification of the mean fluorescence intensity for Nrf2 and HO-1. Data are mean ± SD (n = 3); ns, not significant; *p < 0.05, **p < 0.01, ***p < 0.001, ****p < 0.0001.

To elucidate the mechanisms driving the enhanced closure observed *in vivo* (Figure 6), we examined the histomorphometric and molecular data of the day-14 wounded sections. Masson's trichrome staining showed that the reconstruction of the extracellular matrix was different in various groups. Especially, the wounds treated with PCNZnCy had more and better arranged collagen fibers, which are features of mature granulation tissue 50. However, in the control group and the groups with single components, the deposition of fibrils was in an irregular way (Figure 7A). The quantitative analysis also confirmed this result, indicating a higher collagen volume fraction in the PCNZnCy group, suggesting active new synthesis and maturation of the matrix (Figure 7B). Collectively, this enhanced collagen accumulation clearly indicates vigorous fibroblast activity and coordinated tissue reorganization—events critical to restoring durable skin integrity.

Successful healing hinges on restoring vascularization. To study this, the wound sections were stained with CD31 and VEGF, the main inducer of angiogenesis. In the tissues treated with PCNZnCy, a dense and well-arranged network of CD31-positive microvessels was formed, showing a close co-localization with a higher expression of VEGF (Figure 7C). The fluorescence intensities were measured and it was discovered that both signals were much stronger than those in the corresponding controls (Figure 7D-E), which is consistent with the *in vitro* tube formation results. Thus, the marked neo-vascularization can well clarify the rapid wound healing we have observed, because new capillaries supply the required oxygen and metabolic substrates for repair 51. In conclusion, our *in vivo* findings confirm the pro-angiogenic effects observed in the HUVEC assays (Figure 5).

To confirm the biological significance of the *in vitro* antioxidant results (Figure 4) *in vivo*, we examined the wound sections for the Nrf2/HO-1 pathway. The PCNZnCy-treated samples exhibited strong Nrf2 nuclear translocation and significant HO-1 induction (Figure 7F), with the quantitative fluorescence intensity much higher than that in all other groups (Figure 7G-H). The evident activation of Nrf2/HO-1 in the tissue indicates the antioxidant activity of PCNZnCy, suggesting its long-lasting therapeutic effect even in the complex and changing microenvironment of the wound healing process. By activating this master regulatory pathway, PCNZnCy shifts the wound microenvironment from chronic oxidative stress and inflammation to one conducive to repair. Nrf2 activation not only upregulates HO-1 but also induces downstream cytoprotective genes that collectively reduce inflammation, prevent cellular apoptosis, and promote proliferative signaling, thereby creating a conducive niche for the observed collagen synthesis and angiogenesis. Collectively, PCNZnCy is a versatile wound-healing agent that mitigates infection and ROS while promoting tissue regeneration via collagen deposition and angiogenesis by reprogramming the wound microenvironment. Its ability to combine antibacterial and regenerative functions makes it more than a drug carrier, positioning it as an innovative nanoplatform that targets multiple nodes in the dysfunction of diabetic wound healing and chronic tissue injuries.

### Transcriptomic profiling provides supportive systems-level evidence for PCNZnCy-mediated modulation of the wound microenvironment

**Figure 8:**
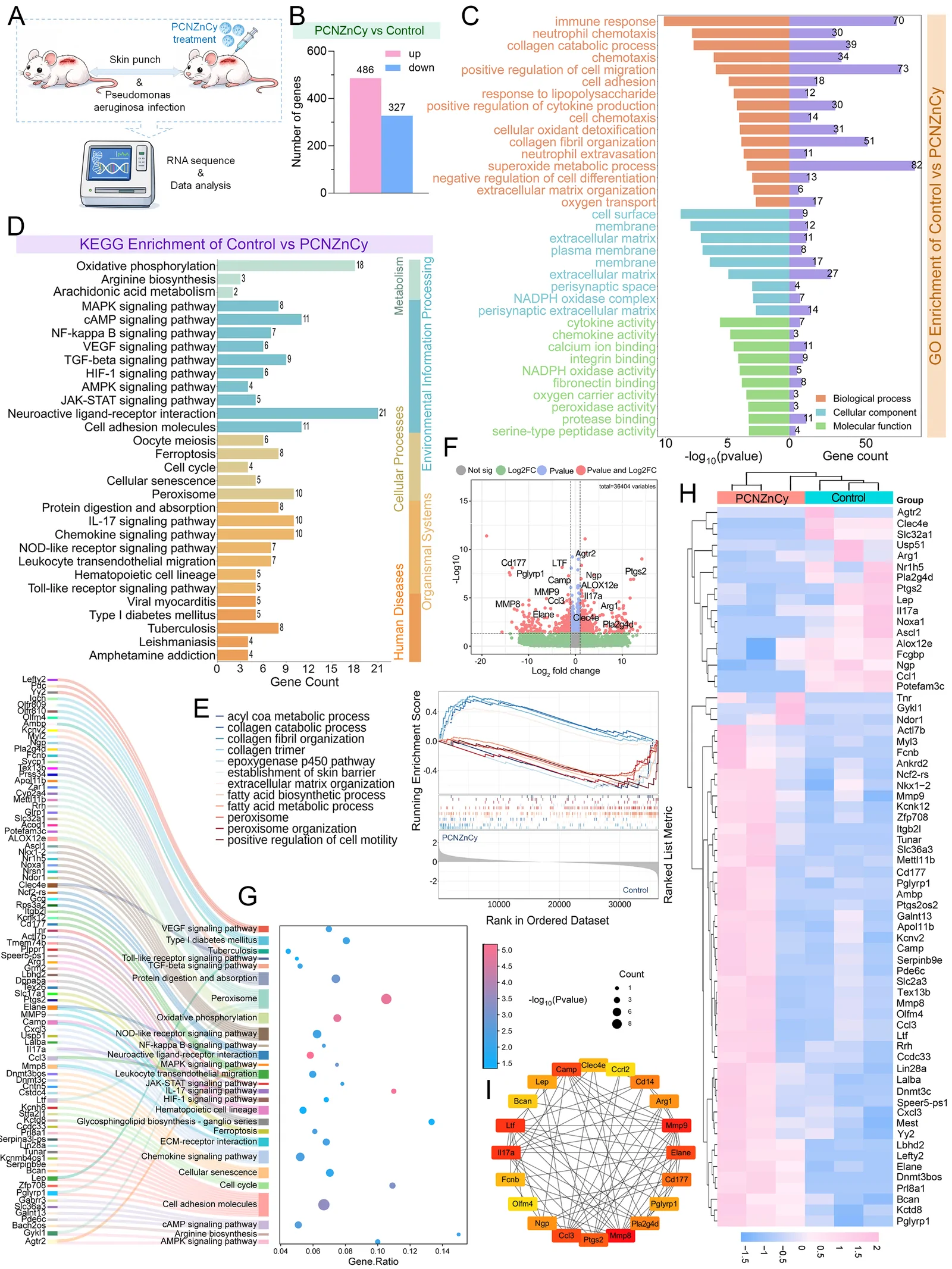
** Transcriptomic profiling uncovers the systemic healing mechanisms of PCNZnCy in diabetic wounds. (A)** Schematic diagram outlining the RNA-seq workflow comparing treated diabetic infected mice against controls. **(B)** Bar plot analysis of DEGs between PCNZnCy-treated and controls. **(C)** GO enrichment analysis of DEGs. **(D)** KEGG pathway enrichment analysis of key signaling pathways. **(E)** GSEA plot enrichment of specific biological gene sets. **(F)** Volcano plot analysis of transcriptional landscape after PCNZnCy treatment. **(G)** Sankey–bubble connecting KEGG enrichment pathways with genes. Bubble diameter scales with gene counts, and color conveys enrichment confidence via -log₁₀(FDR). **(H)** Heatmap of hierarchical clustering to segregate DEGs functionally linked to inflammation, oxidative stress, and tissue repair. **(I)** Protein–protein interaction (PPI) network of key DEGs associated with inflammatory regulation, neutrophil activation, and extracellular matrix remodeling. Data are mean ± SD (n = 3 biological replicates), and DESeq2 was used to identify DEGs.

To gain supportive systems-level insight into the molecular changes associated with PCNZnCy treatment, we performed RNA sequencing on wound tissues from the *PA*-infected diabetic mouse model (Figure 8A). Differential expression analysis revealed pronounced transcriptional modulation in PCNZnCy-treated wounds, with 486 genes significantly upregulated and 327 significantly downregulated relative to controls (Figure 8B). This transcriptomic data indicates a change from stable inflammatory responses to a more effective repairing mechanism. By correlating the differentially expressed genes (DEGs) with Gene Ontology (GO) categories, the differences are related to certain regulatory processes like controlling immune effectors, reducing leukocyte infiltration, preventing oxidative damage, remodeling the extracellular matrix and guiding cell movement (Figure 8C). Furthermore, these molecular markers can reproduce our *in vivo* morphological observations, which links genetic variations with functional results. Hence, PCNZnCy possesses a significant dual role - suppressing harmful inflammatory reactions and simultaneously activating the structural and cellular components necessary for true regeneration.

To clarify the molecular mechanism behind the therapeutic effects of PCNZnCy, we carried out a thorough KEGG pathway enrichment analysis on our transcriptomic data. This analysis revealed marked pathway-level differences: inflammatory pathways, including NF-κB, TNF, and cytokine–cytokine receptor interaction, were associated with the downregulated transcriptional program, whereas ECM–receptor interaction, PI3K–Akt signaling, and focal adhesion were enriched among repair-associated DEGs (Figure 8D, G). The decrease in NF-κB and TNF activities can well explain the reduction of IL-1β, TNF-α and the infiltration of immune cells. On the contrary, the enrichment of PI3K-Akt and ECM-related signals may promote the functions of fibroblasts and endothelial cells 52,53. Therefore, PCNZnCy seems to act by reducing the harmful inflammatory processes while reinforcing the beneficial repair programs. In addition to these specific pathways, a comprehensive transcriptional reorganization was clearly observed. GSEA and volcano plot analyses (Figure 8E–F) revealed widespread and coordinated transcriptional reprogramming across the wound transcriptome. Moreover, the hierarchical clustering of representative genes (Figure 8H) indicated distinct separation between the treatment groups with consistent expression patterns within each group. The high internal consistency strongly confirms the reproducibility of the overall molecular changes and verifies our conclusions based on pathway analysis.

Protein–protein interaction (PPI) network analysis of key DEGs revealed an interconnected module associated with inflammatory regulation, neutrophil activation, and extracellular matrix remodeling, with representative nodes including MMP9, MMP8, ELANE, CD177, CD14, and PTGS2 (Figure 8I). These interconnected genes may collectively regulate inflammatory-cell recruitment and tissue-remodeling processes. The transcriptomic changes, together with the previously observed activation of the Nrf2 pathway (Figure 7F–G), further support the involvement of antioxidant defense *in vivo*. To further examine these pathway-level findings, we performed Western blot analysis of representative proteins associated with NF-κB, Akt, STAT3, and Nrf2/HO-1 signaling. As shown in Figure S10, under H₂O₂-induced oxidative stress, PCNZnCy dose-dependently increased Nrf2 and HO-1 expression while reducing STAT3 abundance, NF-κB phosphorylation, and Akt phosphorylation, providing complementary protein-level support for the pathway-level findings. Additionally, to deepen understanding of the findings, we elucidated the regulatory crosstalk between NF-κB/PI3K-Akt-related pathways and the Nrf2/HO-1 axis using the Nrf2 inhibitor ML385 (Figure S11). Nrf2 inhibition significantly attenuated PCNZnCy-mediated upregulation of Nrf2 and HO-1 and partially reversed the changes in STAT3 abundance, NF-κB phosphorylation, and Akt phosphorylation. These results indicate that the Nrf2/HO-1 axis is a critical node that integrates with broader inflammatory and survival signaling networks modulated by PCNZnCy.

Together, these findings validate transcriptomic analysis and establish PCNZnCy as a multifunctional nanoplatform modulator targeting multiple dysfunctional nodes in diabetic wound healing. By simultaneously reducing hyperinflammation and oxidative stress while promoting matrix formation and pro-regenerative signaling, PCNZnCy modulates the wound environment to promote regeneration. Overall, these transcriptomic results give additional systems-level information which confirm our phenotypic and histopathological findings, but they cannot prove the mechanisms definitively. Further research is needed to directly compare PCN-224, PCN-224(Zn) and free Chrysin, and to apply time-resolved single-cell transcriptomics.

### Biosafety evaluation of PCNZnCy

**Figure 9:**
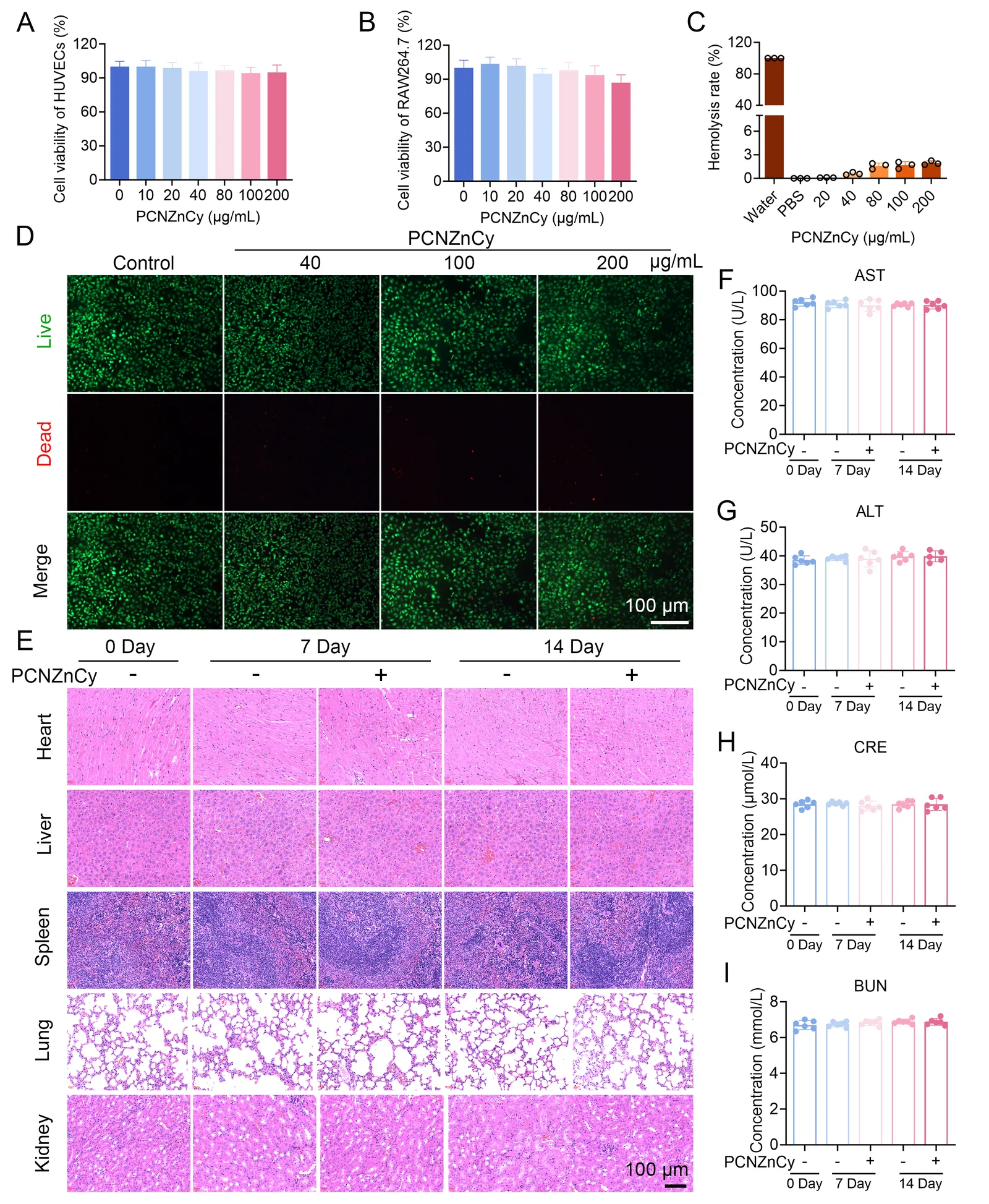
** Biosafety evaluation of PCNZnCy. (A, B)** CCK-8 assay for Cell viability of HUVECs and RAW264.7 cells after 24-hour treatment with different concentrations of PCNZnCy. **(C)** Hemolysis assay of PCNZnCy at various concentrations. **(D)** Live/Dead staining of HUVECs treated with PCNZnCy (40, 100, 200 μg mL**⁻¹**). Green: live cells, red: dead cells. Scale bar: 50 μm.** (E)** H&E images of major organs (heart, liver, spleen, lung, kidney) collected from mice at Days 0, 7, and 14 after PCNZnCy systemic administration. Scale bar: 50 μm.** (F–G)** Serum levels of AST and ALT for liver function assessment. **(H–I)** Serum concentrations of CRE and blood BUN for kidney function evaluation. Data are mean ± SD (n = 3–5).

To evaluate the translational safety of PCNZnCy, we conducted systematic *in vitro* assays to examine its biocompatibility. In the CCK-8 viability assays, the metabolic activities of both HUVECs and RAW264.7 macrophages were above 90% even at the highest concentration tested, 200 μg/mL (Figures 9A-B), indicating a wide therapeutic index. This result was confirmed by the qualitative Live/Dead imaging, where the cultures treated with PCNZnCy exhibited mainly live (green) cells with only a few red-stained dead cells over different doses (Figure 9D). Considering the requirement of blood compatibility for its possible intravenous use, we then measured the hemolytic potential. PCNZnCy showed a hemolysis ratio less than 2% from 5 to 200 μg mL⁻¹, which is well within the acceptable limit of generally less than 5% for biomaterials and nearly equal to the negative PBS control (Figure 9C).

To investigate the systemic toxicity further, a single 50 mg/kg intravenous dose was given to each mouse. The histopathological examination of the major organs (heart, liver, spleen, lung, kidney) which were harvested on the 0th, 7th and 14th days after injection showed no evidence of tissue damage, inflammatory infiltration or abnormal structure in the PCNZnCy-treated group, similar to the control group (Figure 9E). This result is in agreement with the serum data, since the important biomarkers for liver function (ALT, AST) and kidney function (CRE, BUN) maintained their normal physiological levels during the 14 days (Figure 9F-I). Additionally, histopathological evaluation of major organs after local wound application of PCNZnCy revealed no abnormalities, inflammation, necrosis, or tissue damage in treated mice compared to controls (Figure S12). This suggests repeated topical PCNZnCy at a therapeutic dose does not cause systemic organ toxicity.

To strengthen the findings in Figure 9E, we performed a systematic analysis of inflammatory cytokines. Results showed that PCNZnCy did not significantly increase serum levels of TNF-α, IL-1β, and IL-6 on days 0, 7, and 14 after PCNZnCy treatment compared with untreated controls (Figure S13), supporting the absence of inflammatory infiltration in the histopathological examination over the 14-day observation period. The *in vivo* results are particularly significant, as the absence of histological abnormalities in vital organs and the stability of hepatic and renal biomarkers over the 14-day observation period demonstrate that PCNZnCy does not induce acute or sub-acute toxicity under the tested conditions (50 mg/kg, intravenous administration). Given that Zr-based MOFs, such as PCN-224, are known for their chemical stability at physiological pH but degrade in acidic environments 14,31, such as lysosomes or infected wounds, it is plausible that PCNZnCy may undergo gradual degradation (Zr⁴⁺, Zn²⁺, organic linkers) in the body, but direct evidence of its clearance kinetics, metabolic fate, or potential for long-term organ accumulation requires further investigation. Overall, these biosafety data confirm the absence of apparent acute toxicity from PCNZnCy, thereby supporting its continued preclinical development. Beyond that, its combined anti-infective, ROS-scavenging, cytoprotective, and pro-angiogenic properties make it a compelling agent for managing chronic wound care. Yet biodistribution for >14 days and chronic toxicity evaluations are required before future clinical translation.

## Conclusion

This study successfully engineered an innovative, multifunctional, stimuli-responsive dual-metal nanotherapeutic platform that leverages the synergistic potential of its components to combat drug-resistant bacteria. This rationally designed nanocomposite overcomes the limitations of conventional mono-functional MOFs by enabling synchronized antibacterial, antioxidant, and pro-regenerative functions through a pH- and ROS-responsive release mechanism. PCNZnCy demonstrates potent activity against biofilm-embedded multidrug-resistant clinically relevant pathogens, such as MRSA and* PA*, while simultaneously modulating the wound microenvironment through robust free-radical scavenging, mitochondrial protection, and activation of the endogenous Nrf2/HO-1 antioxidant axis. Beyond infection control, PCNZnCy promotes tissue repair in *PA*-infected diabetic wound model by enhancing endothelial cell proliferation, migration, and angiogenesis under oxidative stress, leading to accelerated wound closure, improved collagen deposition, and functional neovascularization in diabetic infected wounds. Systems-level transcriptomic analysis provides supportive evidence that PCNZnCy treatment is associated with a shift from pro-inflammatory to pro-regenerative gene expression profiles, including downregulation of NF-κB- and TNF-related transcriptional programs and enrichment of ECM-remodeling and PI3K–Akt-related gene programs. These findings, together with Western blot and immunofluorescence data, support the involvement of Nrf2/HO-1 activation in the antioxidant and cytoprotective effects.

Critically, PCNZnCy showed excellent biocompatibility and biosafety in both *in vitro* and *in vivo* tests, and no systemic toxicity was detected in mice during the 14-day observation after intravenous injection, which suggests its potential for clinical application. While this safety profile is encouraging, it represents only an initial step toward preclinical development, not an indication of imminent clinical utility. This work introduces a therapeutically effective nanoplatform and highlights several design features, including dual-metal incorporation, flavonoid loading, and stimuli-responsive release, that may inform future MOF development for chronic wound applications. With ongoing optimization and rigorous preclinical validation, PCNZnCy offers a promising research direction for treating multidrug-resistant infected diabetic wounds, though significant work remains before clinical translation can be considered. By combining material innovation with deep mechanistic insights, PCNZnCy offers a promising approach for treating MRSA- and *PA*-infected diabetic wounds, which are multidrug-resistant and clinically challenging. The demonstrated *in vitro* broad-spectrum antibacterial activity and antioxidant properties suggest potential for broader application, but validation in additional *in vivo* models for MRSA infection or polymicrobial and non-diabetic chronic wounds is necessary for future investigation. Further validation of the potential for resistance development under long-term or repeated use warrants future systematic study.

## Materials and Methods

### Reagents and materials

Zirconyl(IV) chloride octahydrate (ZrOCl_2_·8H_2_O, Z164495), benzoic acid (BA, B116255), 5,10,15,20-tetrakis(4-carboxyphenyl)porphyrin (TCPP, P115336), Chrysin (C110079) and N, N-dimethylformamide (DMF, N807505), hydrogen peroxide (H_2_O_2_, H112515), 1-Diphenyl-2-picrylhydrazyl (DPPH, D273092), and 2,2'-azino-bis (3-ethylbenzthiazoline-6-sulfonic acid) (ABTS, A109612) were sourced from Aladdin (Shanghai, China).

Resazurin (R7017) and C11-BODIPY581/591 (SML3717) were obtained from Sigma-Aldrich (USA). Crystal violet staining solution (C0121) and Live/Dead bacterial staining kit with DMAO & PI (C2030S) were obtained from Beyotime (Shanghai, China). Cell counting kit-8 (CCK-8, CA1210), 2’,7’-dichlorofluorescin diacetate (DCFH-DA, D6470), superoxide anion assay kit (BC1295), hydroxyl radical assay kit (BC1235), Calcein-AM/PI stain kit (CA1630), JC-1 mitochondrial membrane potential assay kit (M8650), and SOD/MDA/GPX/CAT determination kits (BC5165/BC0025/BC1195/ BC0205) were purchased from Solarbio (Beijing, China).

Primary Antibodies against Nrf2 (16396-1-AP), HO-1 (10701-1-AP), Cluster of Differentiation 31 (CD31, 11265-1-AP), and Vascular endothelial growth factor (VEGF, 81323-2-RR) were acquired from Proteintech (Wuhan, China). Human umbilical vein endothelial cells (HUVECs) and murine macrophage cells (RAW 264.7) were obtained from the Cell Bank of the Chinese Academy of Sciences (Shanghai, China).

### Preparation and synthesis of PCNZnCy

***Synthesis of PCN-224 NPs:*** PCN-224 nanoparticles were synthesized according to a previously reported method with slight modifications 14. Briefly, ZrOCl₂·8H₂O (300 mg, 0.93 mmol), benzoic acid (2.8 g, 22.93 mmol), and tetrakis(4-carboxyphenyl) porphyrin (TCPP, 100 mg, 0.13 mmol) were dissolved in 100 mL of N, N-dimethylformamide (DMF) under ultrasonication for 20 min to obtain a homogeneous solution. The molar ratio of ZrOCl₂·8H₂O:TCPP:benzoic acid was approximately 7.4:1:181. The mixture was then heated in an oil bath at 90 ± 2 °C under magnetic stirring for 5 h. After cooling to room temperature, the resulting suspension was centrifuged at 12,000 rpm (approximately 13,500 × g) for 20 min. The precipitate was collected and washed three times with DMF to remove unreacted precursors and modulators. The obtained PCN-224 nanoparticles were redispersed in DMF for subsequent Zn incorporation or dried under vacuum at 40 °C for storage.

***Synthesis of PCN-224(Zn) NPs:*** For Zn incorporation, PCN-224 NPs (30 mg) were dispersed in 10 mL of DMF by ultrasonication for 10 min. Zn(NO₃)₂·6H₂O (30 mg, 0.10 mmol) was then added to the suspension, corresponding to a Zn precursor-to-PCN-224 mass ratio of 1:1. The mixture was transferred into a sealed glass reaction vessel and heated at 120 ± 2 °C under magnetic stirring for 12 h to allow Zn coordination within the porphyrinic framework. After cooling to room temperature, the particles were collected by centrifugation at 12,000 rpm, approximately 13,500 × g, for 10 min. The precipitate was washed three times with DMF and three times with deionized water to remove residual Zn salts and solvent. The obtained Zn-doped PCN-224 nanoparticles were denoted as PCN-224(Zn).

***Chrysin loading*** *PCN-224(Zn) NPs **(Chrysin@PCN-224(Zn)) NPs***: To prepare Chrysin-loaded PCN-224(Zn) nanoparticles, PCN-224(Zn) NPs (30 mg) and Chrysin (30 mg, 0.12 mmol) were dispersed in 10 mL of DMF, corresponding to a PCN-224(Zn): Chrysin mass ratio of 1:1. The suspension was stirred at room temperature, 25 ± 2 °C, for 12 h in the dark to facilitate Chrysin loading. The final particles, denoted as PCNZnCy, were collected by centrifugation at 13,500 × g for 10 min. The precipitate was washed three times with DMF and three times with deionized water to remove free or weakly adsorbed Chrysin. Finally, PCNZnCy was dried under vacuum at 40 °C for 12 h and stored in a desiccator until further use.

### Characterization of PCNZnCy

Morphology and structure of PCN-224, PCN-224(Zn), and PCNZnCy were analyzed using scanning electron microscopy (SEM, Zeiss Sigma 300) and transmission electron microscopy (TEM, JEOL F200). The hydrodynamic diameter, particle size distribution, polydispersity index (PDI), and zeta potential were measured using a dynamic light scattering analyzer (Malvern ZetaSizer Nano ZS). Nanoparticle suspensions were prepared by dispersing the samples in deionized water or PBS, followed by brief sonication before measurement. Elemental mapping of C, N, O, Zr, and Zn within the MOFs was performed using High-angle annular dark-field scanning TEM (HAADF-STEM) coupled with energy-dispersive X-ray spectroscopy (EDS). Surface chemical composition and valence states were analyzed by X-ray photoelectron spectroscopy (XPS, Thermo Fisher Scientific K-Alpha system). UV–vis absorption spectra were recorded to confirm the successful encapsulation of Chrysin.

### Drug loading and release behavior of PCNZnCy

Drug loading content (DLC) and encapsulation efficiency (EE) were calculated using a UV–vis standard curve based on Chrysin’s absorbance at 348 nm. The pH/ROS-responsive release capacity of PCNZnCy was conducted in phosphate-buffered saline (PBS) at varying pH (7.4, 6.5, and 5.5) containing H₂O₂ (100 µM, pH 5.5) at 37°C, with UV–vis analysis of supernatant absorbance measured over time.

The release profiles of Zn and Zr from PCNZnCy nanoparticles were determined by ICP-MS. Nanoparticles were dispersed in release media with different physiological/pathological conditions, PBS pH levels (7.4, 6.5, 5.5), and pH 5.5 with H₂O₂, matching the H₂O₂ used in the chrysin assay. Suspensions were placed in dialysis bags, incubated at 37°C with gentle shaking. Aliquots were taken at 0, 2, 4, 6, 8, 10, 12, and 24 hours, then acidified and diluted for ICP-MS. Released Zn and Zr were measured using calibration curves, with blank media for background correction. Total Zn and Zr in nanoparticles were obtained by digesting lyophilized samples with nitric acid.

### Stability assessment of PCNZnCy

Colloidal stability of PCNZnCy was evaluated by suspending the material in deionized water, 0.9% NaCl, PBS, and cell culture medium (90% DMEM + 10% FBS). The initial mixture was homogenized by ultrasonication for 15 minutes. DLS was subsequently used to track particle size over 7 days to detect any agglomeration or sedimentation. Separately, we assessed refrigerated stability across prolonged storage, recording observations at set time intervals.

### Bacterial strains and culture conditions

MRSA (ATCC 43300) and *PA* (ATCC 27853) were purchased from China General Microbiological Culture Collection Center (CGMCC). Cultures were grown for both strains in LB medium at 37 °C under continuous shaking at 200 rpm. To obtain cells in logarithmic growth, bacteria were pelleted by centrifugation (5000 rpm, 5 min), then washed twice with sterile PBS. Finally, bacteria were resuspended to the required concentration just before running antibacterial experiments.

### Antibacterial activity assay

Antibacterial activity of PCNZnCy and the control materials were tested by a standard colony-forming unit (CFU) count method. Specifically, the cultures of MRSA and PA suspension (about 1 × 10⁶ CFU mL⁻¹) along with PCN-224, PCN-224(Zn), free Chrysin or PCNZnCy at the same concentrations were cultured in LB broth at 37 °C for 6 h. The untreated bacteria were used as the negative control. After incubation, serial ten-fold dilutions of each culture were prepared in sterile PBS and 100 μL aliquots were spread onto standard LB agar plates for the determination of the final viable colonies. The bacterial viability was determined by colony counting on LB agar plates after incubating at 37 °C for 18–24 h.

### MIC and MBC determination of PCNZnCy

The minimum inhibitory concentration (MIC) and minimum bactericidal concentration (MBC) of PCNZnCy against MRSA and *PA* were determined using a tube dilution method. Briefly, MRSA and *PA* were cultured overnight in LB medium at 37 °C and diluted with fresh LB medium to approximately 5 × 10⁵ CFU mL⁻¹. PCNZnCy was first dispersed in sterile PBS and then subjected to two-fold serial dilution in sterile centrifuge tubes to obtain a concentration range of 1–512 μg mL⁻¹. Equal volumes of bacterial suspension and PCNZnCy dilution were mixed in sterile centrifuge tubes, with a final volume of 1 mL per tube. Tubes containing bacteria without PCNZnCy served as the growth control, while tubes containing medium alone served as the sterility control. In addition, tubes containing PCNZnCy at the corresponding concentrations without bacteria were included as material background controls. All tubes were incubated at 37 °C for 18–24 h. After incubation, bacterial growth was evaluated by visual inspection of turbidity, compared with the growth control and the corresponding material background control. The MIC was defined as the lowest concentration of PCNZnCy at which no visible bacterial growth was observed.

To determine the MBC, aliquots from tubes showing no visible bacterial growth were spread onto LB agar plates and incubated at 37 °C for 18–24 h. The MBC was defined as the lowest concentration of PCNZnCy that resulted in no visible colony formation or achieved ≥99.9% killing of the initial bacterial inoculum.

### Resazurin metabolic activity assay

Bacterial metabolic activity after treatment was assessed using a resazurin assay. After a 6-hour incubation with the materials, resazurin solution was added to the bacterial suspensions at a final concentration of 10 μg mL⁻¹. The samples were then incubated for 2 more hours at 37 °C in the dark. Metabolic activity was judged visually by the change from blue to pink, and we then measured it precisely by absorbance readings at 570 nm on a usual plate reader. Decrease in absorbance indicates less metabolic production.

### Live/dead bacterial staining

To assess bacterial viability and membrane integrity, the Live/Dead BacLight assay was applied, which colors living cells green by using DMAO and dead cells red with propidium iodide. The treated suspensions were first centrifuged and washed twice with PBS. Then, the samples were stained with both dyes according to the instructions of the manufacturer. After a 15-minute incubation in darkness at room temperature, the fluorescent images were taken by a microscope. Lastly, the micrographs were analyzed with ImageJ to determine the percentages of living and non-living populations in different random areas.

### Crystal violet staining of biofilm formation

To evaluate antibiofilm activity, a crystal violet assay was conducted. The overnight bacterial cultures with concentrations of about 1 × 10⁶ CFU mL⁻¹ were spread into 24-well plates and allowed to adhere for 24 h at 37°C, either with or without the test nanomaterials. After incubation, the planktonic cells were removed by aspiration and each well was washed three times with PBS to detach the loosely attached cells. The remaining sessile communities were fixed with 4% paraformaldehyde for 15 min, and then stained with 0.1% (w/v) crystal violet for 20 min at room temperature. After washing off the unbound stain with distilled water thoroughly, the crystal violet retained in the biofilms was dissolved in 95% ethanol. The absorbance was measured at 570 nm by a plate reader and the biofilm biomass was calculated relative to the control group which is set to 100%.

### Extracellular polysaccharides quantification in biofilms

To first assess how PCNZnCy affects biofilm matrix composition, the amounts of extracellular polysaccharides, which is a major part of EPS, were determined by a usual phenol-sulfuric acid method. For this purpose, static conditions were used to establish the biofilms of MRSA and *PA*. After the growth period, the free-floating cells were separated and the attached biofilms were cleaned with sterile PBS. Then, the formed biofilms were exposed to PCN-224, PCN-224(Zn), Chrysin or PCNZnCy at equal concentrations respectively, while another group remained untreated as a control. Subsequently, the biofilms were washed again and the bacteria were transferred into fresh PBS solution after being scraped. By means of vortexing, the bacteria were separated from the insoluble residues through centrifugation, and the supernatant, which contained the soluble extracellular polysaccharides, was retained. Finally, for colorimetric quantification, aliquots of this supernatant were combined with phenol and concentrated sulfuric acid, then allowed to stand at room temperature to allow full color development. The absorbance was measured at 490 nm using a microplate reader. Glucose was used to generate a standard curve. The extracellular polysaccharide content was normalized to the control group and expressed as relative EPS content (%).

### ABTS•⁺ and DPPH• radical scavenging assay

The antioxidant capacity of PCNZnCy and control samples was measured through standard DPPH and ABTS radical scavenging assays. Briefly, a working solution of the radical cation (ABTS•⁺) was prepared by mixing 7 mM ABTS with 2.45 mM potassium persulfate in distilled water, then incubating in the dark at room temperature for 12–16 h to generate ABTS•⁺ radicals. Before the assay, the ABTS•⁺ solution was diluted with PBS until the absorbance at 734 nm reached approximately 0.70 ± 0.02. Different concentrations of PCNZnCy (10, 20, 35, and 50 μg mL⁻¹) were added to the ABTS•⁺ solution and incubated at room temperature. Absorbance at 734 nm was recorded using a UV–vis spectrophotometer.

To evaluate radical scavenging ability, DPPH was dissolved in ethanol to yield a 0.1 mM solution. PCNZnCy was tested at 10, 20, 35, and 50 μg mL⁻¹ by adding each concentration to the DPPH• mixture. Reactions were left in the dark for 30 min at ambient temperature. Absorbance was recorded at 517 nm on a UV–vis spectrophotometer; the observed decrease reflected scavenging activity.

### Kinetic scavenging analysis of ABTS•⁺ and DPPH• free radicals

For kinetic evaluations, PCNZnCy was combined with its controls at matching concentrations with a freshly prepared ABTS•⁺ working solution, and the absorbance was recorded at 734 nm at regular time intervals over 60 minutes. An identical procedure was carried out again with the DPPH• working solution, and the readout wavelength was fixed at 517 nm. From the obtained data, we plotted the time-dependent decay curves for each radical species. By comparing the resulting profiles, we got a clear understanding of both the initial reaction rates and the total scavenging abilities, which were good references for evaluating the relative performances.

### Cell viability assay (CCK-8)

To assess cytoprotective effects, RAW264.7 macrophages and HUVECs were plated at a density of 5 × 10⁴ cells per well in 96-well plates and allowed to attach overnight. Afterwards, the cells were exposed to equal amounts of PCN-224, PCN-224(Zn), free Chrysin or PCNZnCy for 2 hours. In order to produce oxidative damage, 200 μm H₂O₂ was added for 24 hours, the concentration chosen to simulate the sublethal oxidative stress frequently encountered in chronic diabetic wounds 14. The control wells remained untreated. Then, 10 μl of CCK-8 solution was added to each well and the plates were incubated at 37 °C for 1–2 hours. The optical density at 450 nm was measured with a standard microplate reader, and relative cell viabilities were calculated by normalizing each treatment group's absorbance to that of the untreated control.

### Statistical analysis

GraphPad Prism 8.0 (GraphPad Software, La Jolla, CA, USA) was used for statistical analyses. All data are shown as the mean ± standard deviation (SD) throughout, except where explicitly indicated. For direct comparisons between two experimental groups, the unpaired two-tailed Student’s t-test was applied. For comparison of three or more groups, one-way ANOVA with Tukey’s post hoc test was used. Significance was we set at *p < 0.05* and denote *p < 0.05, **p < 0.01, ***p < 0.001, and ****p < 0.0001 in figures. Each experiment was independently repeated at least 3 times.

## Supplementary Material

Supplementary figures and methods.

## Data Availability

Data will be made available on request.
