# Supplementary material for: Dual-metallic porphyrinic MOFs with pH/ROS-responsive release for antibacterial and antioxidant therapy of diabetic infected wounds
Source: Theranostics. 2026 Jul 29;16(15):8521–45. doi: 10.7150/thno.135365 (PMC13549140; doi:10.7150/thno.135365)
Supplement: Supplementary file 1 [file thnov16p8521s1.pdf]

Supporting information

Supplementary figures and legends

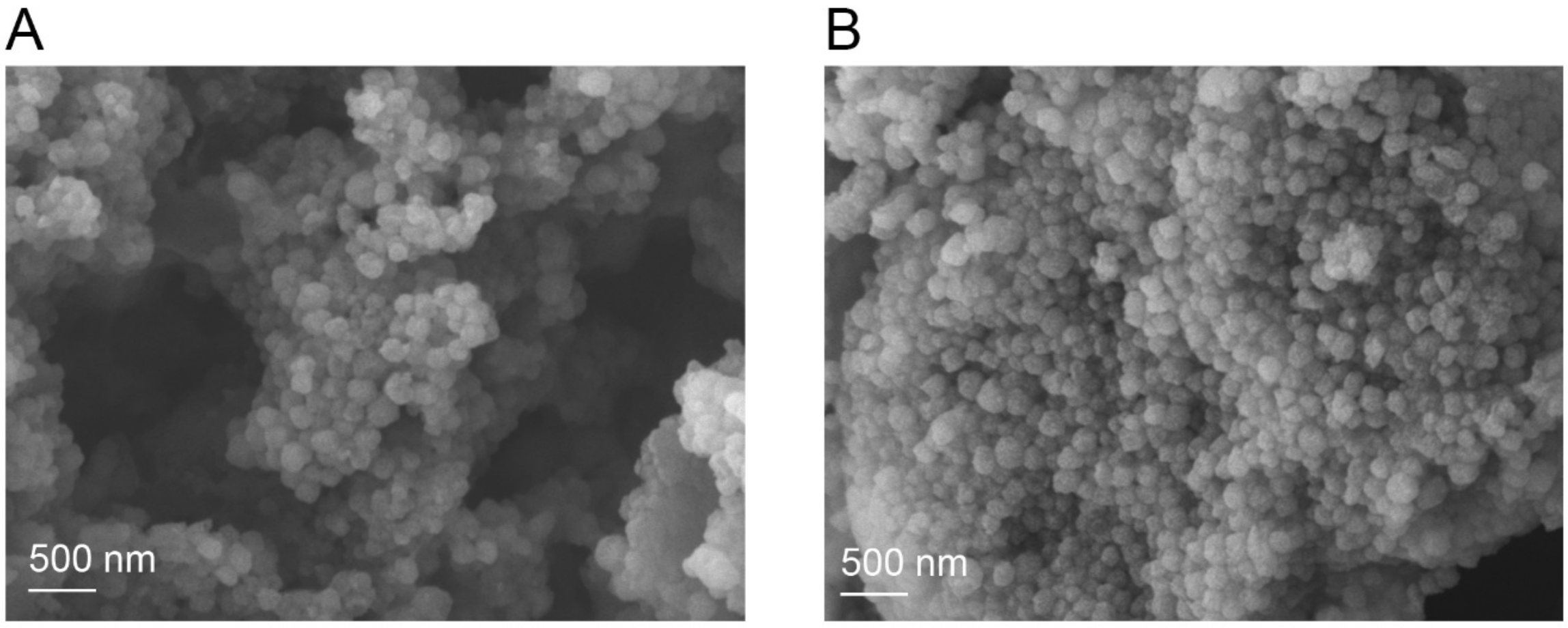

Figure S1. (A, B) Representative SEM images of PCN-224 (A) and PCN-224(Zn) (B). Scale bar: 200 nm

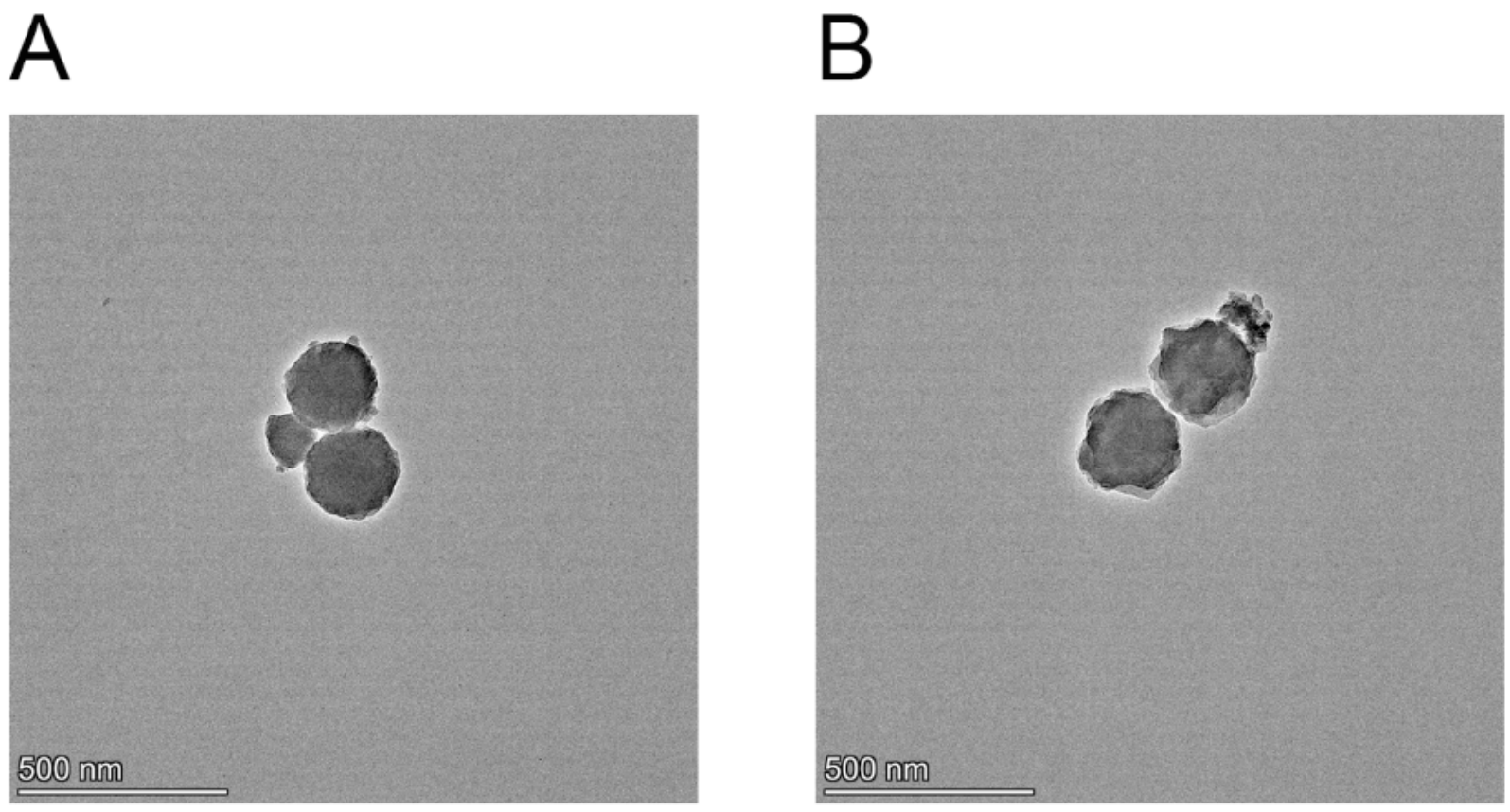

Figure S2. (A, B) Representative TEM images of PCN-224 (A) and PCN-224(Zn) (B). Scale bar: 500 nm.

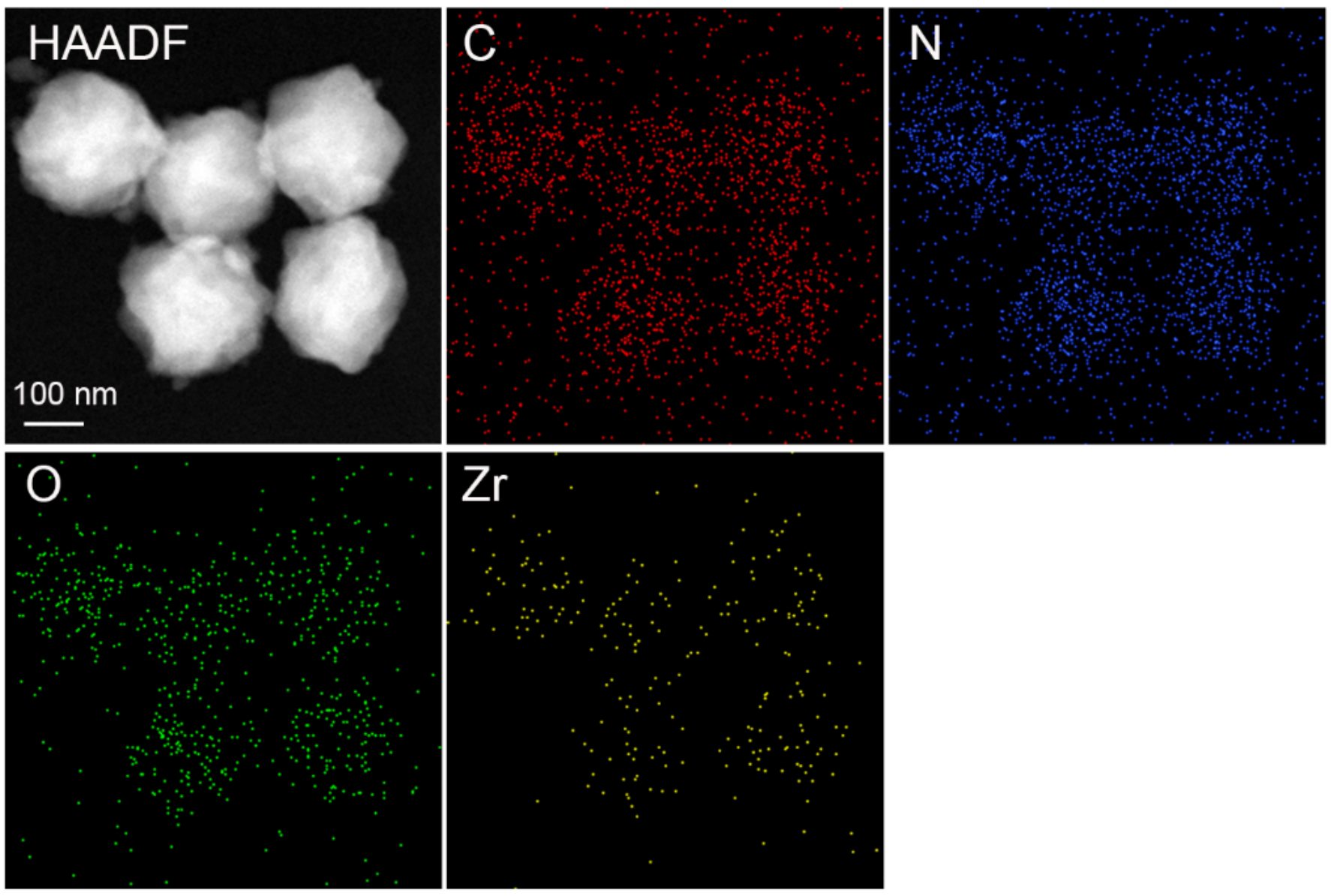

Figure S3. HAADF-STEM elemental mapping images of PCN-224. Scale bar: 100 nm

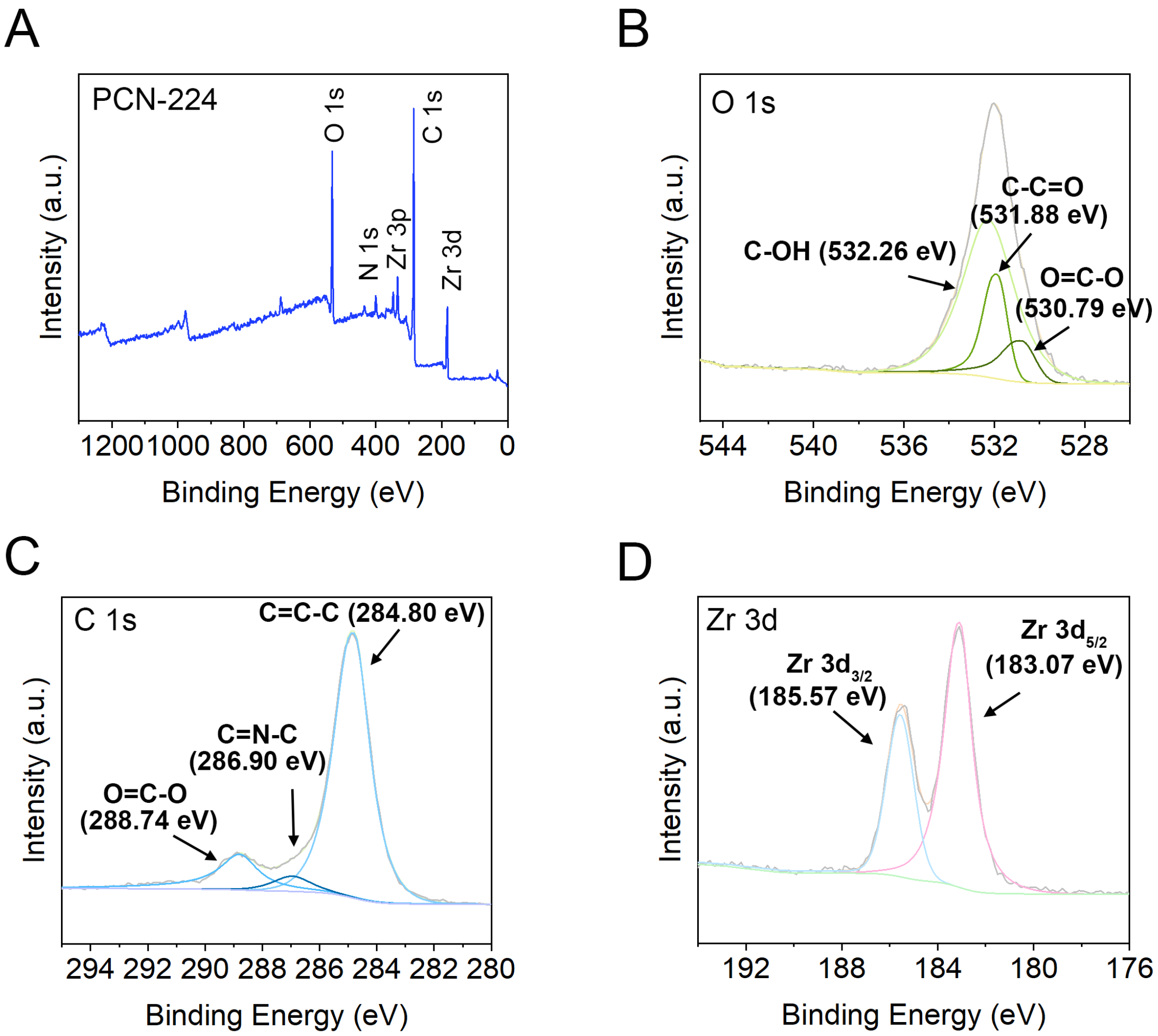

Figure S4. (A) XPS full spectrum of PCN-224. (B-D) High-resolution XPS C 1s, O 1s, and Zr 3d spectra of PCN-224, respectively.

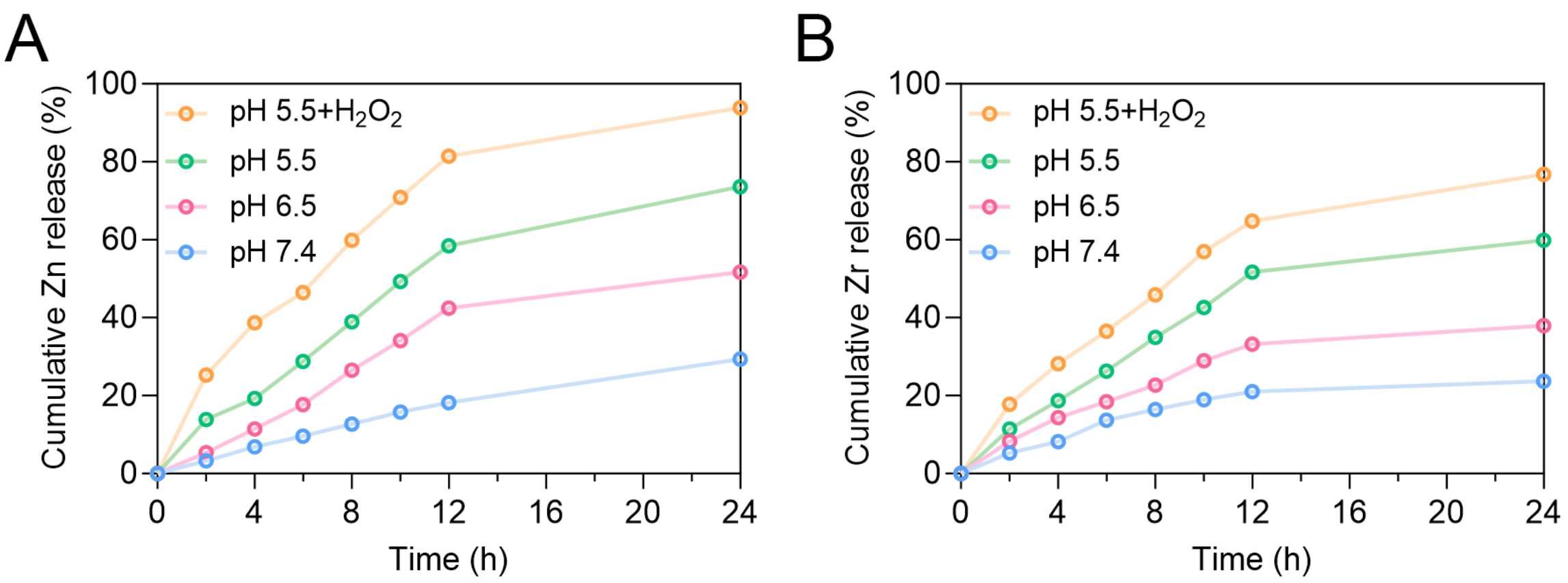

Figure S5. (A) Cumulative Zn release and (B) cumulative Zr release from PCNZnCy under different conditions, including pH 7.4, pH 6.5, pH 5.5, and pH 5.5 + H_2O_2.

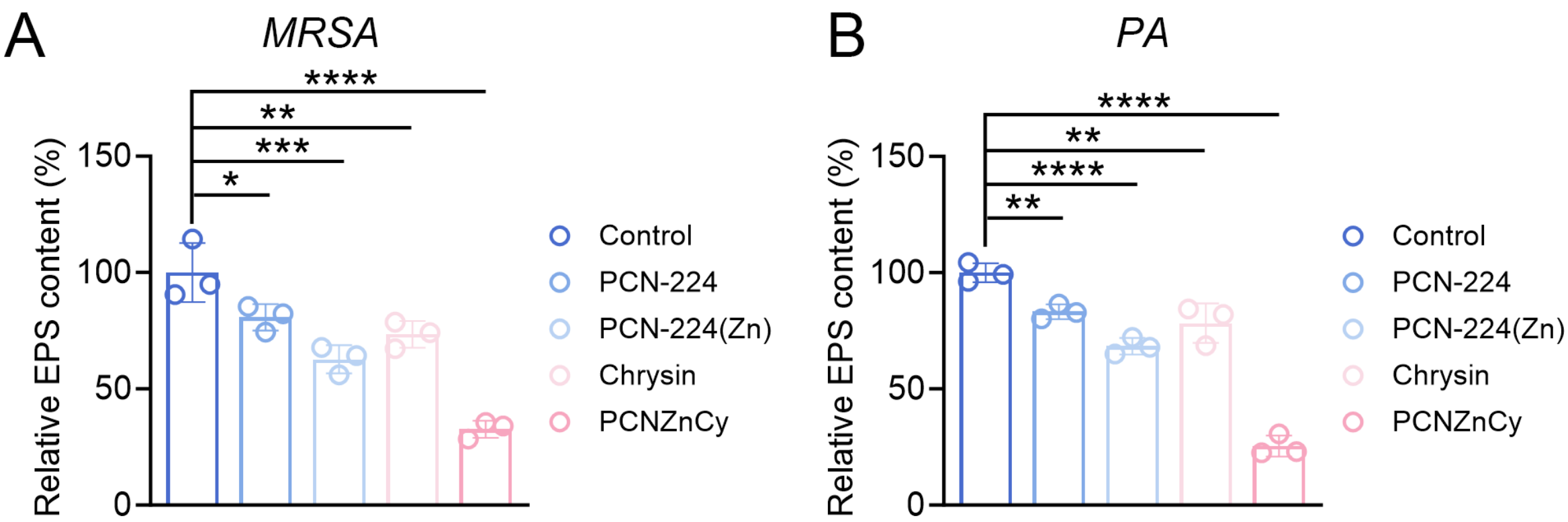

Figure S6. (A, B) Relative extracellular polysaccharide content in MRSA and P. aeruginosa biofilms after different treatments. Data are presented as mean ± SD. *$p < 0.05$, **$p < 0.01$, ***$p < 0.001$, ****$p < 0.0001$.

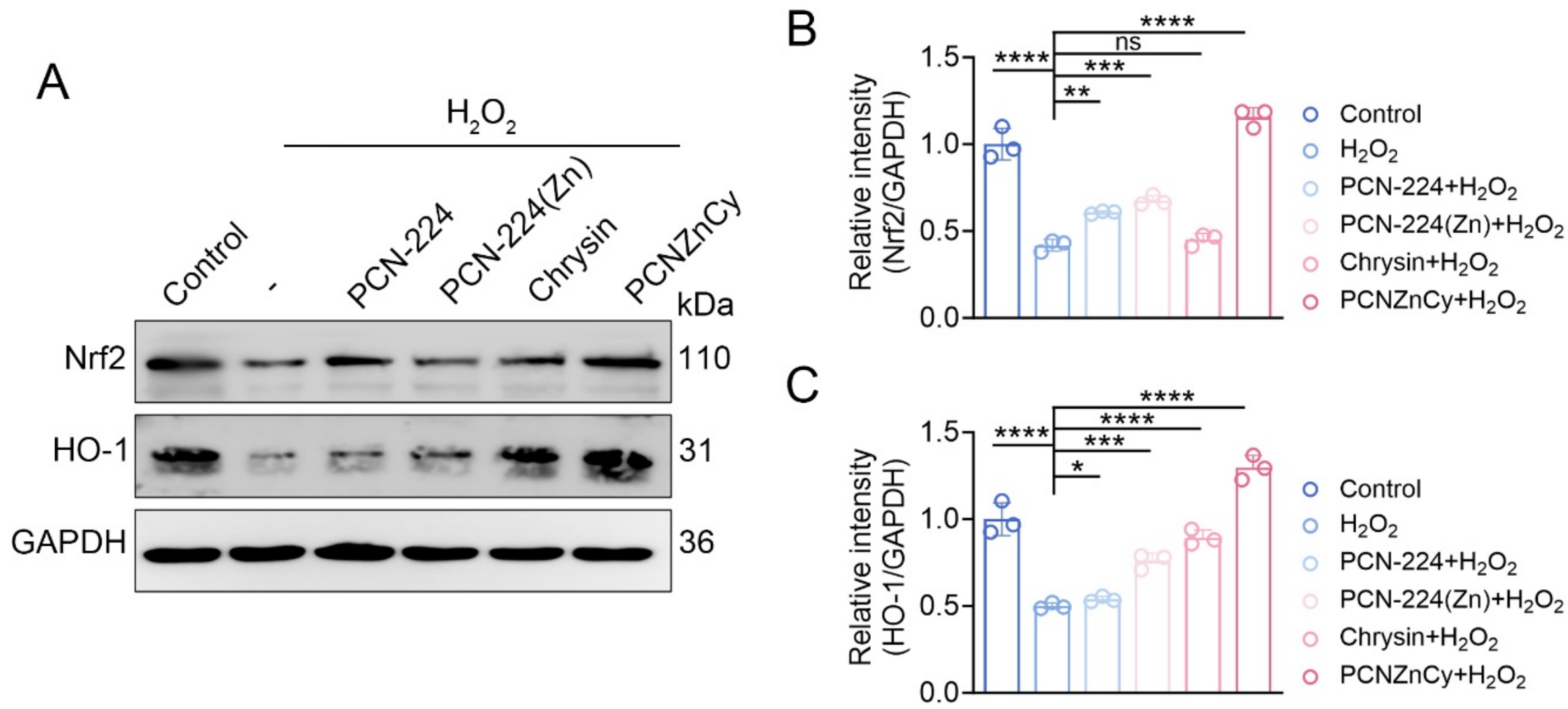

Figure S7. (A) Representative Western blot bands of Nrf2 and HO-1 in cells treated with H_2O_2 alone or in combination with PCN-224, PCN-224(Zn), Chrysin, or PCNZnCy. GAPDH was used as the loading control. (B, C) Quantitative analysis of Nrf2 and HO-1 protein expression

normalized to GAPDH. Data are presented as mean ± SD, n = 3. ns, not significant; **p* < 0.05*, ***p < 0.01, ***p < 0.001, ****p < 0.0001*.

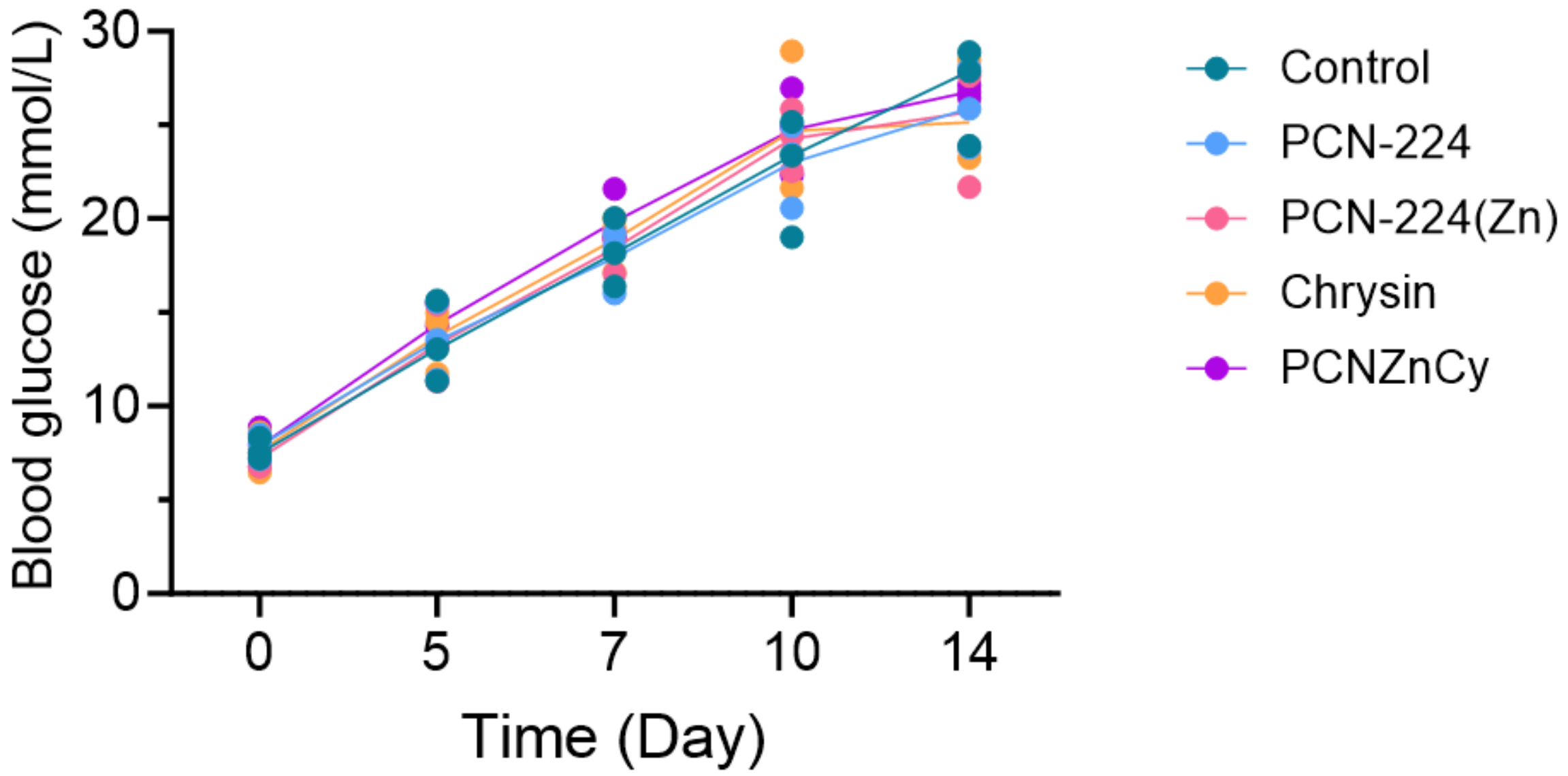

Figure S8. Fasting blood glucose levels were monitored during the establishment of the diabetic mouse model and subsequent treatment period. Data are presented as mean ± SD.

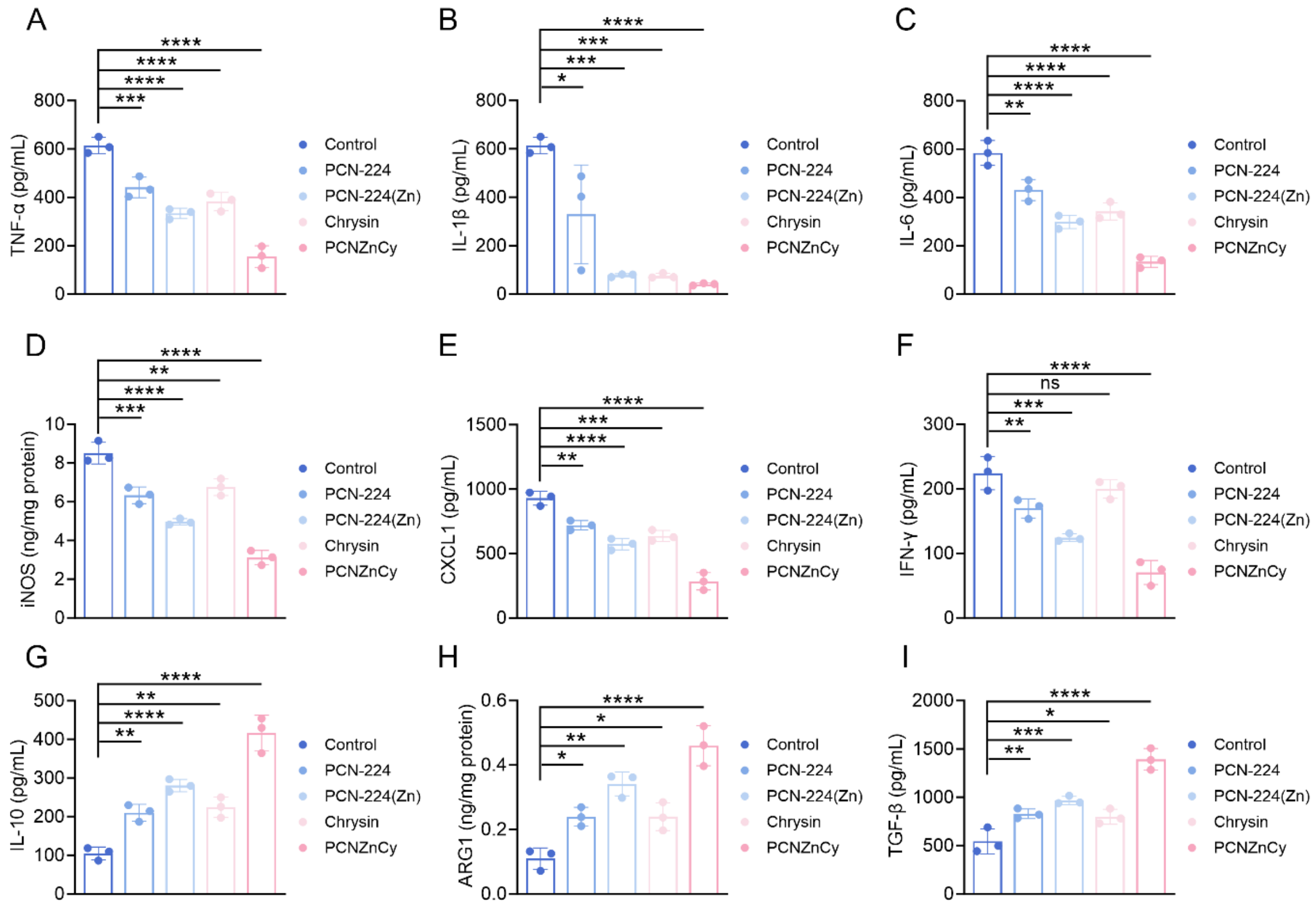

Figure S9. (A–C) Levels of pro-inflammatory cytokines TNF-α, IL-1β, and IL-6 in wound tissues after different treatments. (D–F) Levels of inflammation-associated mediators iNOS, CXCL1, and IFN-γ. (G–I) Levels of anti-inflammatory and tissue repair-related markers IL-10, ARG1, and TGF-β. Data are presented as mean ± SD, n = 3. ns, not significant; **p* < 0.05*, ***p < 0.01, ***p < 0.001, ****p < 0.0001*.

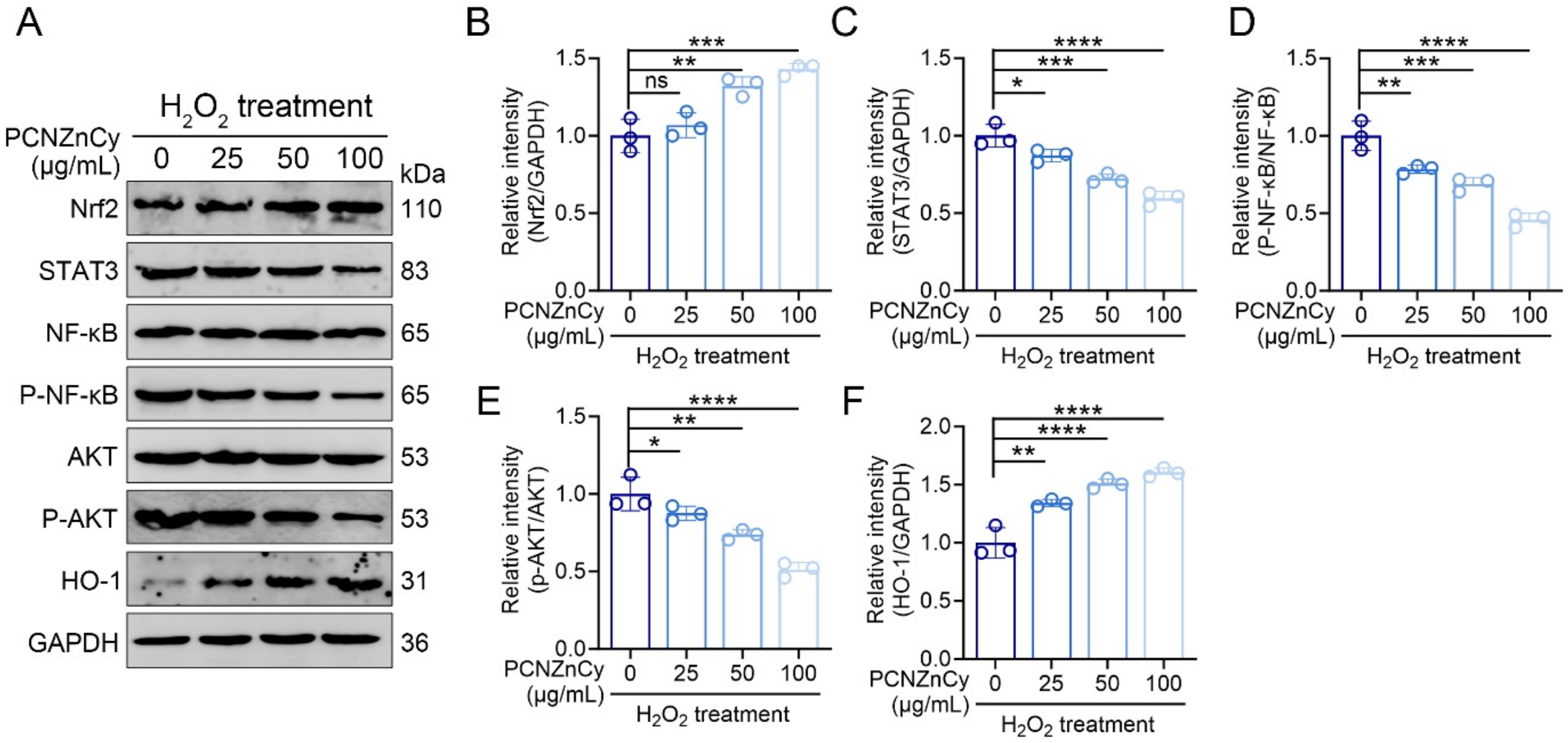

Figure S10. PCNZnCy regulates Nrf2/HO-1-, NF-κB-, STAT3-, and Akt-related signaling under H_2O_2-induced oxidative stress. (A) Representative Western blot bands of Nrf2, STAT3, NF-κB, p-NF-κB, Akt, p-Akt, HO-1, and GAPDH in cells treated with different concentrations of PCNZnCy under H_2O_2 stimulation. (B–F) Quantitative analysis of Nrf2, STAT3, p-NF-κB/NF-κB, p-Akt/Akt, and HO-1 expression levels. Data are presented as mean ± SD. ns, not significant; *$p < 0.05$, **$p < 0.01$, ***$p < 0.001$, ****$p < 0.0001$.

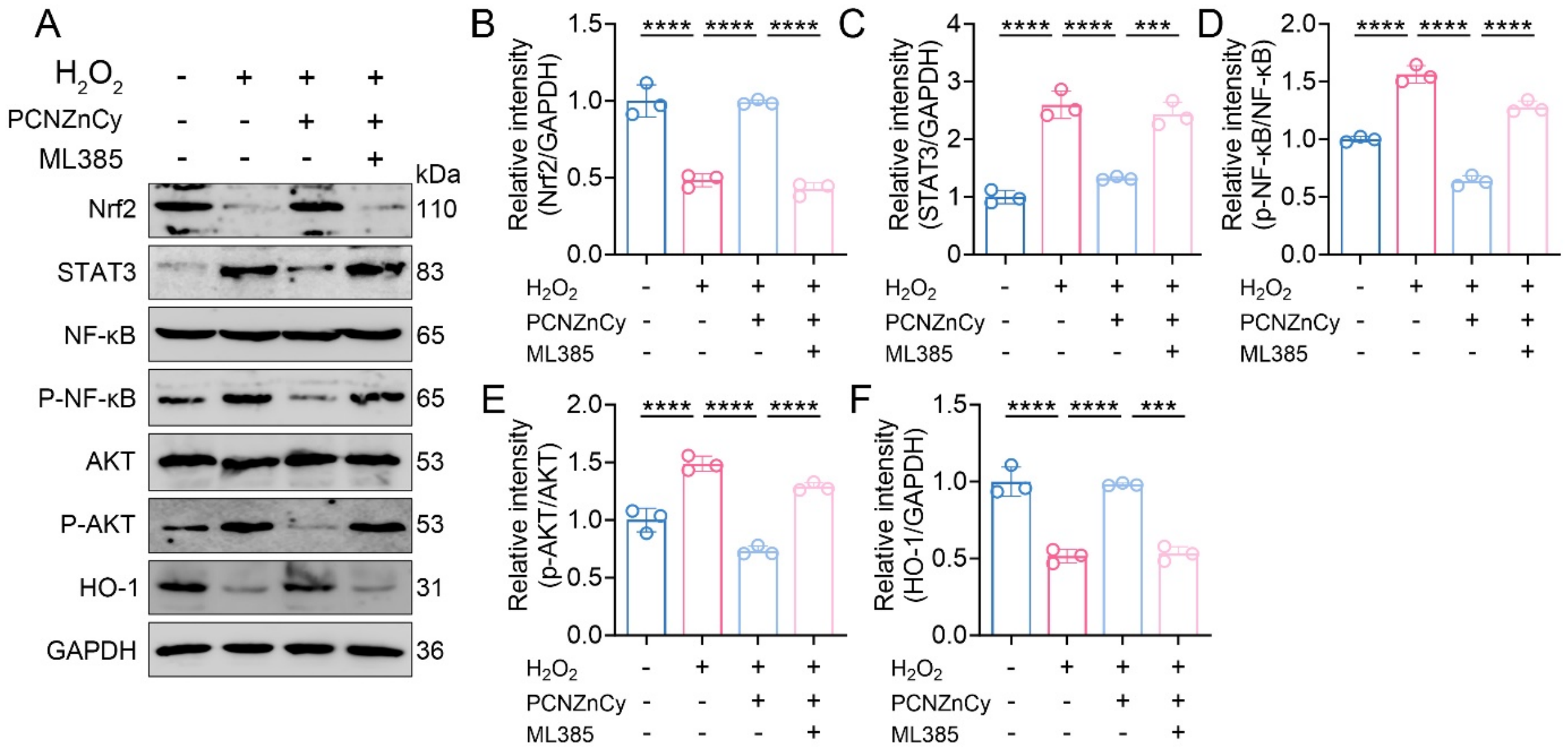

Figure S11. Nrf2 inhibition partially affects PCNZnCy-mediated regulation of NF-κB, STAT3, Akt, and HO-1 signaling. (A) Representative Western blot bands of Nrf2, STAT3, NF-κB, p-NF-κB, Akt, p-Akt, HO-1, and GAPDH in cells treated with H_2O_2, PCNZnCy, and the Nrf2 inhibitor ML385. (B–F) Quantitative analysis of Nrf2, STAT3, p-NF-κB/NF-κB, p-Akt/Akt, and HO-1 expression levels. Data are presented as mean ± SD. ***$p < 0.001$, ****$p < 0.0001$.

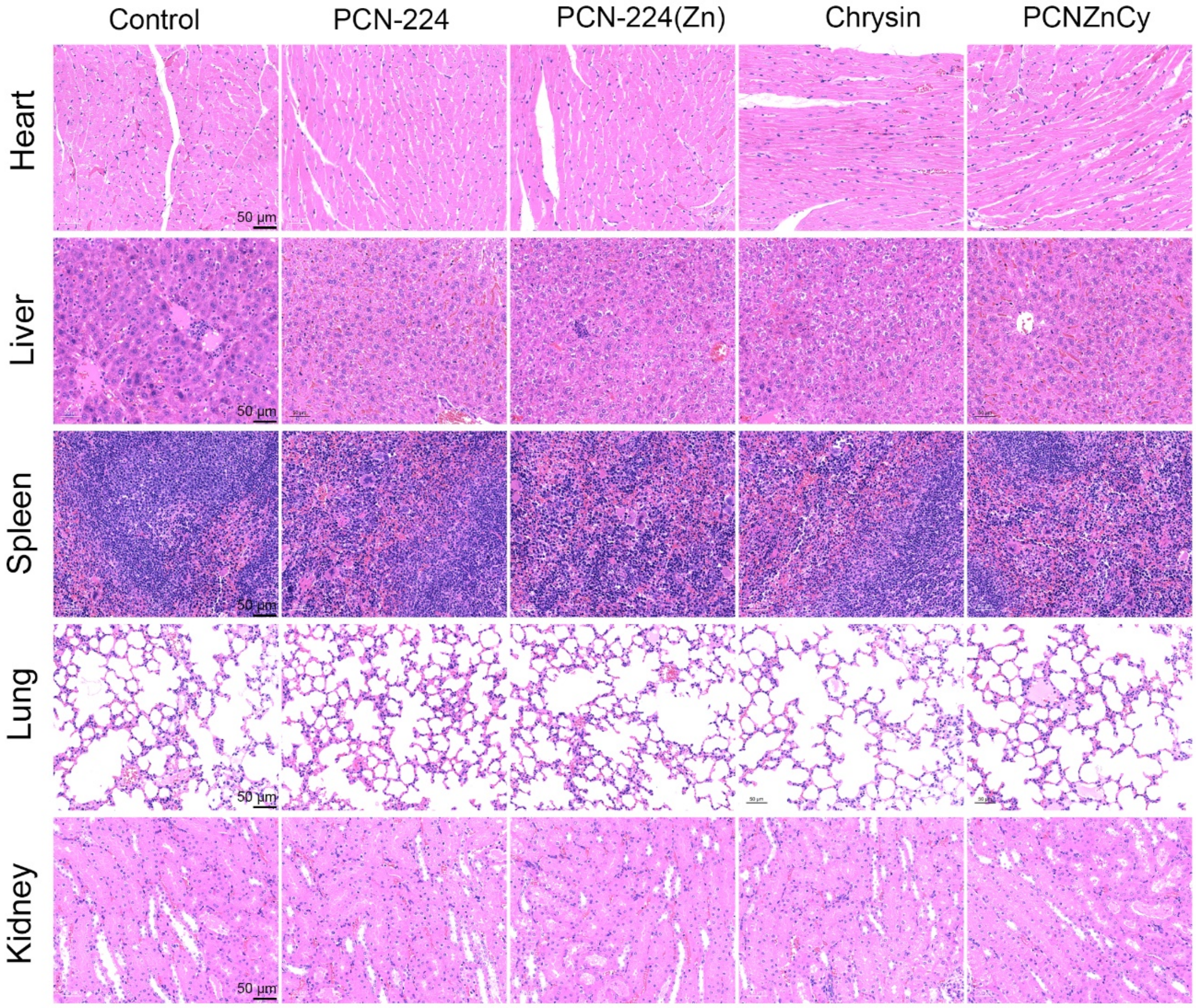

Figure S12. Representative H&E staining images of the heart, liver, spleen, lung, and kidney collected from mice treated with Control, PCN-224, PCN-224(Zn), Chrysin, or PCNZnCy. Scale bar: 50 μm.

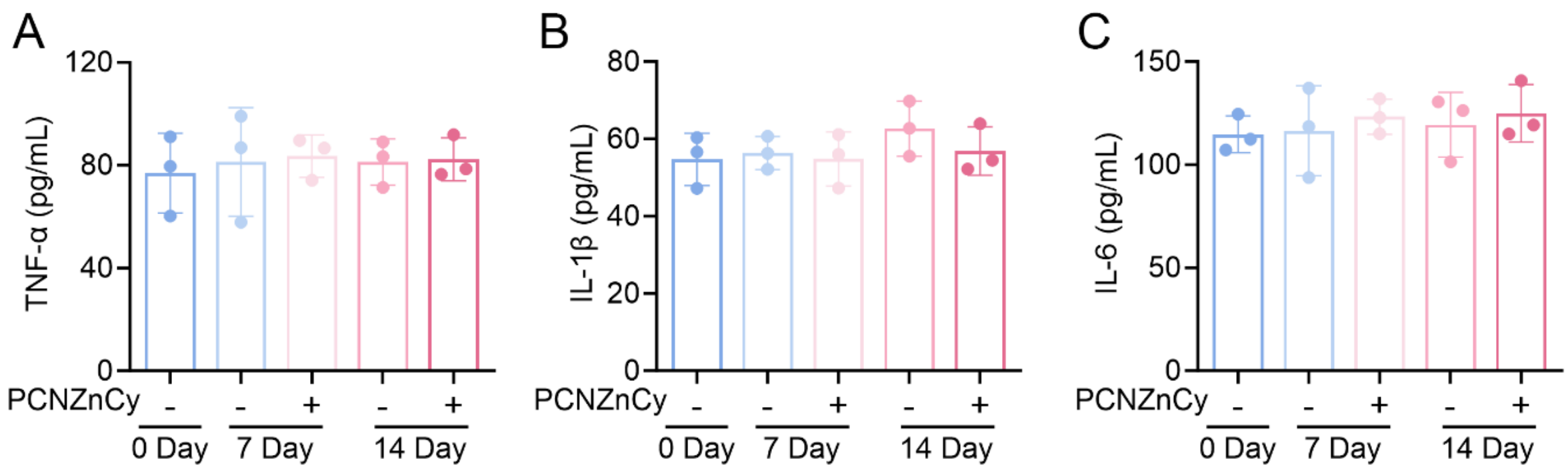

Figure S13. (A–C) Serum levels of TNF-α, IL-1β, and IL-6 in mice at day 0, day 7, and day 14 after treatment with or without PCNZnCy. Data are presented as mean ± SD, n = 3.

Table S1. Hydrodynamic diameter, PDI and zeta potential of different nanoparticles.

Sample	Hydrodynamic diameter (nm)	PDI	Zeta potential (mV)
PCN-224	145.6 ± 4.2	0.128 ± 0.015	−24.3 ± 1.8
PCN-224(Zn)	181.9 ± 5.6	0.152 ± 0.018	−16.7 ± 1.5
PCNZnCy	209.4 ± 6.3	0.176 ± 0.021	−20.8 ± 1.6

Table S2. The minimum inhibitory and bactericidal concentration (MIC/MBC) of PCNZnCy against *S. aureus* and *P. aeruginosa*.

Bacterial Strain	MIC (μg mL^{-1})	MBC (μg mL^{-1})
MRSA (ATCC 43300)	64	128
P. aeruginosa (ATCC 27853)	32	64

Supplementary experimental section

1. Methods

1.1. Measurement of intracellular oxidative stress-related enzyme activities

malondialdehyde (MDA) concentrations and the activities of antioxidant enzymes, including glutathione peroxidase (GPx), catalase (CAT), and superoxide dismutase (SOD), were determined using specific commercial assay kits. Briefly, RAW264.7 cells were harvested, lysed with the supplied buffer, and centrifuged at 12,000 rpm for 10 min at 4 °C. The resulting supernatant was analyzed using commercial kits as per the manufacturer's protocols. Total protein concentrations in the lysates were quantified using a BCA assay kit, and enzyme activities were normalized to protein content. Results were expressed as nmol or U per mg protein.

1.2. Intracellular ROS detection

Intracellular reactive oxygen species (ROS) were quantified using the fluorescent probe DCFH-DA. RAW264.7 cells and HUVECs were seeded in confocal dishes or 24-well plates and subjected to the indicated treatments. After treatment, cells were washed twice with PBS and stained with 10 μM DCFH-DA for 30 min at 37 °C in the dark. Excess probe was removed by washing with PBS. Fluorescence images were acquired with a fluorescence microscope, and the mean fluorescence intensity was measured using ImageJ.

1.3. Mitochondrial membrane potential (ΔΨm) analysis using JC-1 staining

Mitochondrial membrane potential was assessed using the JC-1 fluorescent probe. Treated cells were rinsed with PBS and subsequently stained with a JC-1 working solution for 20 minutes at 37°C in the dark. After incubation, cells were washed with JC-1 staining buffer and observed under a fluorescence microscope. JC-1 aggregates (red fluorescence) indicate intact mitochondrial membrane potential, whereas JC-1 monomers (green fluorescence) indicate mitochondrial depolarization. The red-to-green fluorescence intensity ratio was calculated to track changes in the mitochondrial membrane potential.

1.4. Lipid peroxidation detection using BODIPY-C11 staining

Lipid peroxidation was detected by means of the BODIPY-C11 reagent. The cells were firstly cleaned and immersed in PBS, then incubated with 5 μM probe for 30 min at 37 °C in darkness. After staining, the cells were rinsed with PBS again and observed through a fluorescence microscope. The green fluorescence is produced by the oxidized form of BODIPY-C11 whereas the reduced form gives off red fluorescence. The degree of lipid

peroxidation was statistically evaluated by measuring the fluorescence intensity ratio of green to red using ImageJ.

1.5. EdU staining for Cell proliferation

Cell proliferation was evaluated using a 5-ethynyl-2′-deoxyuridine (EdU) incorporation kit following the manufacturer's protocol. HUVECs were cultured overnight in 24-well plates at a density of 5×10^4 cells/well. Before inducing oxidative stress with 200 μM H_2O_2 for 24 h, Cells were pretreated for 2 h with PCN-224, PCN-224(Zn), free Chrysin, or PCNZnCy. Untreated cells were used as the control group. After the H_2O_2 challenge, cells were incubated with EdU working solution according to the manufacturer's instructions for 2 h at 37 °C, then fixed with 4% paraformaldehyde and permeabilized with 0.5% Triton X-100. Briefly, incorporated EdU was detected via Click-iT chemistry, all cell nuclei were counterstained with DAPI, images were captured using a standard fluorescence microscope, and the final proliferation rate was quantified as the percentage of EdU-positive nuclei.

1.6. Scratch wound healing assay

The migratory capacity of HUVECs was evaluated using a standard scratch assay. HUVECs were seeded into 6-well plates and allowed to reach near confluence. A uniform scratch was created in the cell monolayer using a sterile 200 μL pipette tip. After a PBS wash to remove cellular debris, Cells were then incubated with serum-free medium containing H_2O_2 (200 μM) in the presence or absence of PCN-224, PCN-224(Zn), free Chrysin, or PCNZnCy. Images of the wound area were captured immediately at time zero and 48 h post-scratch using an optical microscope. The migration rate was calculated as the percentage of wound closure relative to the initial wound area using ImageJ software.

1.7. Tube formation assay

Endothelial tube formation was used as a measure of angiogenesis potential. Matrigel was thawed overnight at 4 °C. Pre-chilled 96-well plates were coated with 50 μL of Matrigel per well and then incubated at 37 °C for 30 min to solidify. Subsequently, HUVECs were seeded at a density of 2×10^4 cells per well onto the Matrigel-coated wells and treated with H_2O_2 (200 μM) in the presence or absence of PCN-224, PCN-224(Zn), free Chrysin, or PCNZnCy. After 6-hour incubation at 37 °C, tube-like structures were observed and imaged using an inverted microscope. Quantitative analysis, including branch count and total branch length, was performed using ImageJ.

1.8. Transwell migration assay

A Transwell chamber assay (8 μm pore size) was used to evaluate migration. HUVECs in serum-free medium were seeded into the upper chambers in serum-free medium. The lower chambers received complete medium containing 200 μM H_2O_2 and the relevant treatments.

After incubation for 24 h at 37 °C, non-migrated cells on the membrane's upper surface were gently removed with a cotton swab. Migrated cells on the lower surface were fixed with 4% paraformaldehyde, stained with crystal violet, and photographed under an optical microscope. The number of migrated cells per field was quantified using ImageJ.

1.9. Diabetic wound model and PA infection

Animals and Diabetic Model Induction: Male ICR (6–8 weeks old, 20–25 g) were maintained under standard housing conditions with *ad libitum* access to food and water. Diabetes was induced via intraperitoneal injection of streptozotocin (STZ, 50 mg kg^{-1} in citrate buffer, pH 4.5) administered daily for five consecutive days. Age-matched control mice received an equal volume of citrate buffer. One week following the final injection, fasting Blood glucose levels were measured using a glucometer. Mice with fasting blood glucose levels >16.7 mmol/L^{-1} for two consecutive measurements were considered diabetic and used for subsequent experiments. mice satisfying specific inclusion criteria Were enrolled for the wound infection and treatment studies. All procedures received approval from the Jiangsu Normal University Animal Care and Use Committee (JSNU-IACUC-2026006) and complied with national guidelines for the humane care of laboratory animals.

Wound Creation and Infection Protocol: After the diabetic condition was confirmed, the mice were anesthetized using isoflurane, the back part was shaved and disinfected with 75% ethanol. A circular wound, 8 mm in diameter, was made with a sterile biopsy punch, until we reached the panniculus carnosus. In order to prove that the blood sugar remained high, the blood glucose was measured every week during the experiment with a glucometer. For the infection test, PA was grown in LB broth at 37°C for one night, then the cells were collected by centrifugation, washed and resuspended in sterile PBS. At once after the wound was cut, 20 µL of this bacterial suspension containing 10^7 CFU was applied onto the wound surface, which was not covered intentionally to allow bacteria to grow.

Treatment Administration: During a 14-day period, the interventions were mainly performed with a two-day interval. Before using the PCNZnCy, it was freshly prepared by dissolving it in sterile PBS at a concentration of 2 mg/mL (based on the weight of the MOF scaffold) and sonicated for 15 minutes. Then, 50 µL of this solution was injected directly onto the wound with a sterile pipette and spread evenly over the entire wound area. For the control groups, the same volume of sterile PBS or single-component formulations (PCN-224, PCN-224(Zn) or free chrysin) was applied, adjusting the concentrations so that they were equal to 100 µg of MOF scaffold or the corresponding chrysin content in PCNZnCy. These treatments were given once every two days from day 3 to day 13 after the injury. After each administration,

the wounds were covered with sterile gauze and a transparent occlusive dressing to keep the moisture and prevent external contamination. In order to achieve direct and fair comparisons among the different groups, the treatments were standardized according to their chrysin content. Furthermore, the tolerance of the subjects was observed during the experiment by recording their body weights at certain time points.

Wound Monitoring and Analysis: Wounds were photographed on day 0, 3, 5, 7, 9, 11 and 14 with a uniform imaging apparatus, and the areas of wounds were then traced and measured in ImageJ.

1.10. Quantification of bacterial burden in wound tissues

At the final stage of the experiment, the wounded tissues were aseptically removed and their weights were recorded immediately. Then, each sample was homogenized in sterile PBS with a tissue homogenizer. From the homogenates, tenfold serial dilutions were made in PBS and 100 µl of each dilution was spread onto LB agar plates. After incubating the plates at 37 °C for 18-24 hours, the number of visible bacterial colonies was counted directly and presented as colony-forming units (CFU) per wound.

1.11. H&E and Masson's trichrome staining of wound tissues

After the treatment was over, the wounded tissues, including the newly grown skin and the adjacent normal edges, were collected and immediately fixed in 4% paraformaldehyde at room temperature for 24 hours. Then, these specimens were dehydrated with ethanol and cleared with xylene before being embedded in paraffin. The blocks obtained were cut into a thickness of 4–5 µm on a microtome. For the routine histopathological examination, the sections were stained with hematoxylin and eosin according to the standard procedures, paying special attention to re-epithelialization, formation of granulation tissue, inflammatory cell infiltration and the overall structure of the tissue. Adjacent sections were stained with Masson's trichrome, following the manufacturer's instructions, to evaluate collagen deposition and extracellular matrix remodeling. With this approach, collagen fibers appeared blue, while muscle and cytoplasm appeared red. Histological images were captured using a light microscope under identical imaging parameters. Quantitative analysis of collagen volume fraction in Masson-stained sections was performed in ImageJ by calculating the ratio of collagen-positive area to total wound area.

$$\text{Collagen Volume Fraction (CVF)} = \frac{A_{Collagen}}{A_{total}} \times 100\%$$

Where $A_{collagen}$ is the total area stained blue (collagen-positive) in the Masson's trichrome image, and A_{total} is the total wound area (or the reference tissue area) in the same image field.

1.12. ELISA analysis

The levels of inflammatory and repair-related mediators in wound tissues and serum were measured using commercial ELISA kits according to the manufacturers' instructions. Briefly, wound tissues were collected, weighed, homogenized in cold PBS or lysis buffer containing protease inhibitors, and centrifuged at 12,000 rpm for 10 min at 4 °C. The supernatants were collected for ELISA detection of TNF-α, IL-1β, IL-6, CXCL1, IFN-γ, IL-10, TGF-β, iNOS, and ARG1.

For serum analysis, blood samples were collected at the indicated time points and centrifuged at 3,000 rpm for 10 min to obtain serum. Serum TNF-α, IL-1β, and IL-6 levels were measured by ELISA. Absorbance values were recorded at 450 nm using a microplate reader, unknown concentrations were derived from the standard curves, and all samples were assayed in triplicate.

1.13. Immunofluorescence staining

After deparaffinization and rehydration by using graded ethanol, heat induced antigen retrieval was done in citrate buffer (pH 6.0). The wound sections were first blocked with 5% BSA for one hour at room temperature, then incubated at 4°C overnight with rabbit anti-CD31 and mouse anti-VEGF. After three PBST washes, the sections were labeled with secondary antibodies conjugated with different fluorescent dyes and stained with DAPI. The images were taken by confocal laser scanning microscopy. To evaluate the results, the mean fluorescence intensity and the percentage of positive area per high power field were measured using ImageJ, which enables the determination of the number of $CD31^+$ vessels and VEGF expression.

1.14. RNA sequencing and transcriptomic analysis

For the purpose of transcript analysis, the wound tissues taken from diabetic mice which were infected by PA and were treated with either PCNZnCy or saline were extracted. These samples were immediately frozen in liquid nitrogen and kept at -80°C until molecular studies. Total RNA was isolated by using TRIzol reagent according to the standard protocol of the manufacturer. The concentration and purity of each extract were measured by a NanoDrop spectrophotometer and the RNA integrity was checked by an Agilent 2100 Bioanalyzer. Then, only those samples with RNA integrity number (RIN) larger than 7.0 were selected for constructing libraries. The RNA-seq libraries were established with a common poly-A mRNA enrichment kit and sequenced in pair-end mode on an Illumina platform. After sequencing, the raw data were processed by a strict quality-control pipeline to remove adapters and low-quality

sequences. Finally, the clean reads were aligned to the mouse reference genome by a conventional alignment algorithm. The expression levels were then normalized and presented as fragments per kilobase of exon per million mapped reads (FPKM).

Differentially expressed genes (DEGs) between the PCNZnCy treated and control groups were determined by the conditions of $|\log_2(\text{fold change})| \geq 1$ and an adjusted p-value less than 0.05. In order to investigate the functional association, Gene Ontology (GO) and KEGG pathway enrichment analyses were carried out on the DEG set. Besides, Gene Set Enrichment Analysis (GSEA) was used to detect wider transcriptional changes and pathway regulation, without depending on fixed thresholds. For the purpose of representation, Sankey-bubble plots were developed to illustrate the relationships between genes and pathways. Hierarchical clustering heatmaps were utilized to summarize the expression patterns of genes involved in inflammation, oxidative stress and tissue repair. Furthermore, protein-protein interaction networks were constructed from public databases and analyzed to identify the key nodes which may be responsible for the biological effect of PCNZnCy.

1.15. Evaluation of biosafety *in vitro* and *in vivo*

To evaluate the cytocompatibility of PCNZnCy on both HUVECs and RAW264.7 macrophages, the cells were seeded in 96-well plates and allowed to attach overnight. The next day, they were exposed to different concentrations of PCNZnCy (0, 10, 20, 40, 80, 100 and 200 μg mL^{-1}) for a total of 24 hours. The metabolic activity was determined by using the CCK-8 reagent according to the kit's instructions and the absorbance was measured at 450 nm, normalizing the results to the untreated controls. In order to assess the membrane integrity more directly, live/dead staining was conducted on the cells treated with 40, 100 and 200 μg mL^{-1}. These samples were simultaneously stained with Calcein-AM and propidium iodide and observed under a common fluorescence microscope.

Hemocompatibility was tested by a hemolysis assay with freshly obtained mouse red blood cells (RBCs). After separation and washing, RBCs were re-suspended and treated with different concentrations of PCNZnCy (20–200 μg mL^{-1}) for 2 h at 37 °C. Distilled water and PBS acted as positive and negative controls, respectively. Subsequently, the samples were centrifuged and the absorbance at 540 nm of the supernatant was measured to determine the hemolysis rate. To evaluate the *in vivo* biocompatibility, healthy mice were injected intravenously with the same therapeutic dose of PCNZnCy as used in the wound-healing experiments. Blood samples were taken from the retro-orbital plexus at the beginning and on the 7th and 14th day after injection for serum biochemical analysis. Liver function was assessed by measuring serum alanine transaminase (ALT) and aspartate transaminase (AST) levels, while kidney function was

checked by means of creatinine (CRE) and blood urea nitrogen (BUN) tests using commercial kits. At the end of the experiment, major organs including the heart, liver, spleen, lungs and kidneys were excised, fixed in 4% paraformaldehyde, embedded in paraffin, sliced and stained with hematoxylin and eosin (H&E). The histological sections were observed under a light microscope to detect any morphological alterations, tissue injuries or inflammatory changes.

1.16. AI tool disclosure

ChatGPT was only employed to improve the English text during the revision of the manuscript. It did not participate in the experiment planning, data acquisition, statistical examination, figure construction or the result interpretation. All AI-assisted content was checked and approved by the authors.
